# Foundation species stabilize an alternative eutrophic state in nutrient-disturbed ponds via selection on microbial community

**DOI:** 10.3389/fmicb.2024.1310374

**Published:** 2024-04-02

**Authors:** Aditya Jeevannavar, Anita Narwani, Blake Matthews, Piet Spaak, Jeanine Brantschen, Elvira Mächler, Florian Altermatt, Manu Tamminen

**Affiliations:** ^1^Department of Biology, University of Turku, Turku, Finland; ^2^Department of Aquatic Ecology, Eawag, Dübendorf, Switzerland; ^3^Department of Fish Ecology and Evolution, Eawag, Kastanienbaum, Switzerland; ^4^Department of Evolutionary Biology and Environmental Studies, University of Zurich, Zürich, Switzerland

**Keywords:** eutrophication, foundation species, microbial community, amplicon sequencing, *Dreissena*, *Myriophyllum*

## Abstract

Eutrophication due to nutrient addition can result in major alterations in aquatic ecosystem productivity. Foundation species, individually and interactively, whether present as invasive species or as instruments of ecosystem management and restoration, can have unwanted effects like stabilizing turbid eutrophic states. In this study, we used whole-pond experimental manipulations to investigate the impacts of disturbance by nutrient additions in the presence and absence of two foundation species: *Dreissena polymorpha* (a freshwater mussel) and *Myriophyllum spicatum* (a macrophyte). We tracked how nutrient additions to ponds changed the prokaryotic and eukaryotic communities, using 16S, 18S, and COI amplicon sequencing. The nutrient disturbance and foundation species imposed strong selection on the prokaryotic communities, but not on the microbial eukaryotic communities. The prokaryotic communities changed increasingly over time as the nutrient disturbance intensified. Post-disturbance, the foundation species stabilized the prokaryotic communities as observed by the reduced rate of change in community composition. Our analysis suggests that prokaryotic community change contributed both directly and indirectly to major changes in ecosystem properties, including pH and dissolved oxygen. Our work shows that nutrient disturbance and foundation species strongly affect the prokaryotic community composition and stability, and that the presence of foundation species can, in some cases, promote the emergence and persistence of a turbid eutrophic ecosystem state.

## 1 Introduction

Ponds and shallow lakes have been known to respond nonlinearly to nutrient inputs, manifested as transitions between macrophyte-rich clear waters and phytoplankton-rich turbid waters (Scheffer et al., 1993; Janssen et al., 2014). Transitions to a turbid state can lead to harmful algal blooms and dead zones as well as have adverse effects on human health and reduce economic outcomes (Council of the European Communities, 1991; Ibelings et al., 2008), and are therefore generally considered undesirable (OSPAR Commission, 2005). Nutrient reduction alone may not reverse the ecosystem from an undesirable turbid state to a desirable clear state, but coupling the reduction with food web manipulation could accelerate the reversal (van Donk and Gulati, 1995). The nonlinearity of the transitions could therefore partly result from the impacts of key ecosystem species such as macrophytes and grazers (Scheffer et al., 1993; van Donk and Gulati, 1995). Narwani et al. (2019) studied the impacts of two such species on artificial pond ecosystems as nutrient levels increased. However, this study could not account for the complete complexity of the aquatic microbiomes since only microscopy was used for species identification. Here, we investigated this experimental setting further using metabarcoding.

In many ecosystems, a single species exerts strong control over community structure and modulates the dynamics of fundamental ecosystem processes, further referred to as a “foundation species” (Dayton, 1972; Ellison et al., 2005). Some early theoretical studies investigating the impact of biodiversity on the stability of communities or ecosystem functioning suggested that communities with fewer interacting species were more stable (May, 1973), while many current theoretical and empirical studies indicate the opposite (Cottingham et al., 2001; Tilman et al., 2006; Isbell et al., 2009). In certain cases, foundation species can have negative effects on the ecosystem like facilitating and stabilizing turbid eutrophic states (Altieri et al., 2010; Angelini et al., 2011), for instance by selecting for taxa that have increased resistance or susceptibility to eutrophic conditions (Lehman and Tilman, 2000). Foundation species can also affect the ecosystem stability more directly by regulating the fluxes of energy and nutrients in the system or through positive interactions with other taxa in the system (Stachowicz, 2001; Ellison et al., 2005; Schöb et al., 2012). Different foundation species can affect the ecosystems in various ways—for instance, macrophytes affect nutrient cycling, sedimentation levels, create habitats for micro- and macroinvertebrates, and reduce the disruption and resuspension of sediment and sediment-nutrients via mixing and turbulence (Bergström et al., 2000; Declerck et al., 2007; Thomaz and da Cunha, 2010). The presence of macrophytes increases habitat complexity and the abundance and diversity of fishes, invertebrates, and microorganisms in water bodies (Thomaz and da Cunha, 2010). The mere physical presence of macrophyte structures can increase the taxonomic richness of phytoplankton and zooplankton, and live macrophytes may also act via other mechanisms, such as the release of allelochemicals (Declerck et al., 2007). Zebra mussels, on the other hand, increase water clarity and concentrate soluble nitrogen and phosphorus obtained from the planktonic food web and shunt it to littoral food webs (Caraco et al., 1997; Makarewicz et al., 2000; Altieri and Witman, 2006; Strayer, 2009). Zebra mussels are highly invasive, provide habitat and food for invertebrates in the lake shores they occupy, and can have short-term direct and long-term indirect effects on aquatic ecosystems (Strayer, 2009; Karatayev et al., 2015). They are known to cause withering of planktonic food webs, flourishing of littoral food webs, eruptions of toxic phytoplankton, and increasing concentrations of soluble nitrogen and phosphorus (Karatayev et al., 2015). Further, they can cause a sharp fall in the populations of consumers, benthic animals and large zooplankton (Karatayev et al., 2015).

In addition to the foundation species, the ecological stability of freshwater systems is affected by variables such as environmental stochasticity, species richness and community composition and nutrient levels (Grimm and Wissel, 1997; Pennekamp et al., 2018). The ecosystem’s “stability” response to an external disturbance can itself be multi-dimensional (Donohue et al., 2013; Hillebrand et al., 2018) comprising resistance—ability to withstand disturbance (Donohue et al., 2016), recovery—return to initial structure and function (Grafton et al., 2019), resilience—recovery speed after disturbance is removed (Pimm, 1984), and temporal stability—invariability to change both during presence and absence of disturbance, either from one timepoint to another or across a series of timepoints (Hillebrand et al., 2018).

In this study, we focused on temporal stability by observing the rate of change of community composition from its previous state, before, during, and after a nutrient disturbance. The experiment was conducted in an artificial freshwater pond system and the ecosystem dynamics were observed over a period of 13 months, focussing on the individual and interactive impacts of two foundation species on microbial community structure (both prokarya and eukarya). The foundation species used were the Eurasian macrophyte, *Myriophyllum spicatum* (hereafter “*Myriophyllum*”), and the zebra mussel*, Dreissena polymorpha* (hereafter “*Dreissena*”). We tested the hypothesis that the foundation species differentially affect the response of the microbial community structure to changes in the environment, and differentially impact abiotic system variables indirectly via the microbial community.

Previous work on this same experiment investigated how *Myriophyllum* and *Dreissena* affect both abiotic (turbidity, dissolved oxygen etc.) and certain biotic (trait evenness, abundance of cyanobacteria, diatoms etc.) ecosystem properties over an 8 months period (Narwani et al., 2019). It was reported that both foundation species individually reduced the standing stock of phytoplankton biomass while the rate of the biomass increase in response to nutrient addition was higher when the foundation species were present individually and lower when both were present together (Narwani et al., 2019). This was taken as a demonstration that the co-occurrence of foundation species can lead to unexpected ecological outcomes. However, the previous study did not investigate the complete complexity of the microbial community which could explain some of the unanticipated effects of the foundation species’ interactions. Here we present additional investigations of the impacts of the foundation species via the microbial community using new data generated from the amplification of 16S and 18S ribosomal DNA. This enables a deeper analysis of the changes in the total microbial community (both prokarya and eukarya), which was not possible based on the prior data.

## 2 Materials and methods

### 2.1 Experimental setup

We used 16 artificial ponds, of 15 m^3^, in a full-factorial manipulation of the presence and absence of *Dreissena* and *Myriophyllum*, with 4 replicates for each treatment combination. The ponds are situated at the Eawag research facility in Dübendorf, Switzerland (47.4038° N, 8.6098° E). These identical ponds are in-ground and fibreglass-lined. Each features a shallow (0.5 m) and a deep end (2.0 m). To the ponds containing the foundational species, either 100 live macrophytes (mean dry biomass = 19.84 g), 100 live mussels (mean soft tissue dry biomass = 632.67 mg), or a combination of both were added. Henceforth, ponds with only *Dreissena* are referred to as *Dreissena* (or D), only *Myriophyllum* as *Myriophyllum* (or M), both as *Dreissena* + *Myriophyllum* (or MD), and neither as Control (or C).

**Figure 1:**
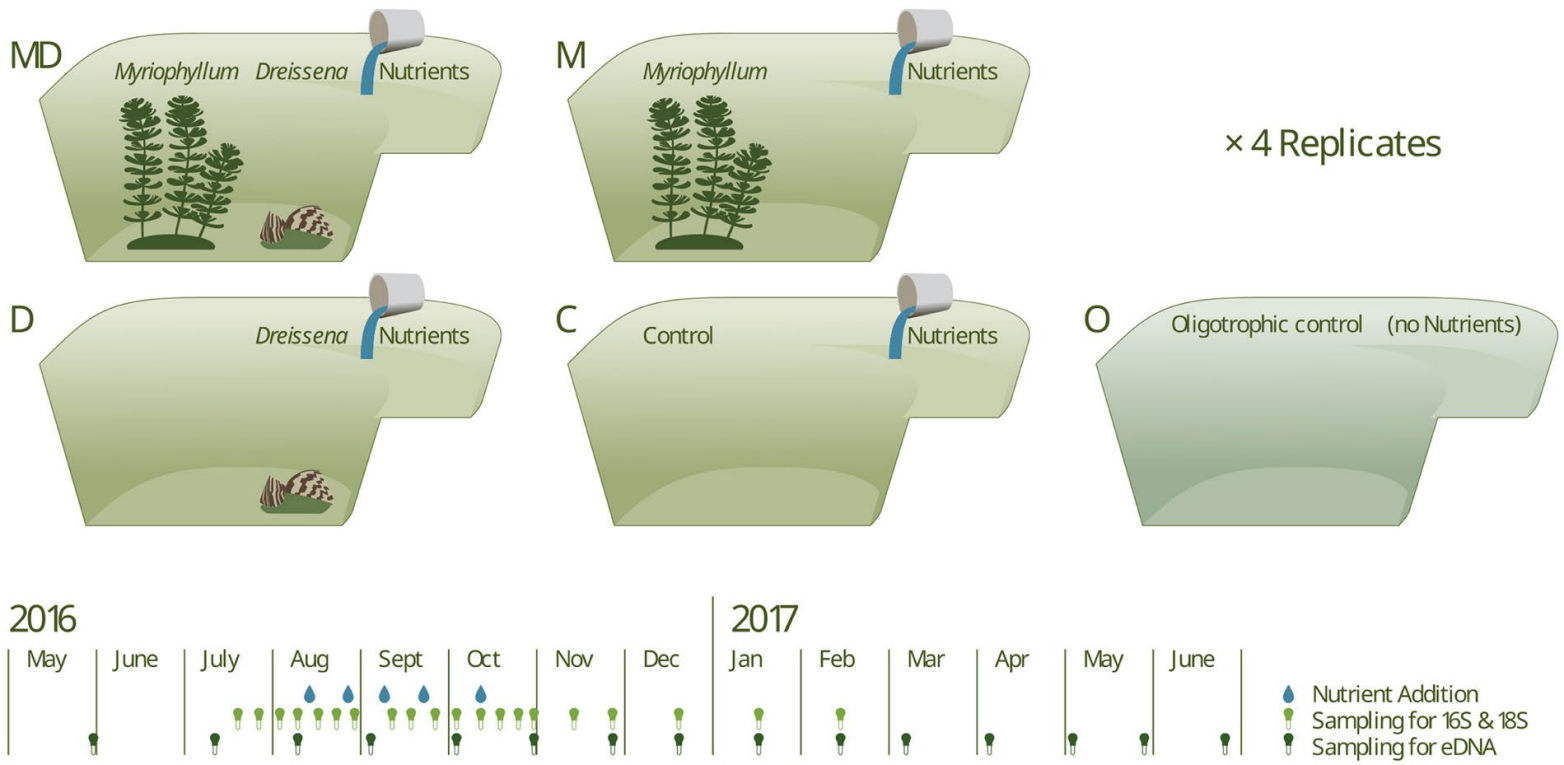
The experimental setup consisted of 16 artificial ponds in four experimental combinations and four additional ponds as oligotrophic controls (i.e., no nutrient additions). In a full-factorial design, ponds contained the foundation species, *Dreissena* and *Myriophyllum* respectively, complemented with an oligotrophic control. Five nutrient additions, of increasing intensity, in the form of KNO_3_ and K_2_HPO_4_ were made for all but the oligotrophic ponds. Sampling for 16S and 18S amplicon sequencing was done weekly and sampling for eDNA (sequencing targeting COI gene) was done monthly.

The experimental ponds were set up in June 2016, and nutrient additions began in Sept 2016. Each of the 16 ponds received increasing doses of nutrients in the form of KNO_3_ and K_2_HPO_4_ five times at 2–2.5 weeks intervals. The five phosphorus additions were of the concentrations 10, 20, 30, 40, and 50 μg l^−1^, and the corresponding nitrogen additions were 32 times of the phosphorus additions, i.e., double the Redfield ratio since nitrogen can escape the system via denitrification. Four additional ponds were used as “oligotrophic” controls (O) that received neither of the foundation species nor any nutrient additions. For a graphical summary of the experimental setup, refer to Figure 1, and for detailed methods regarding the pond experiment, please refer to Narwani et al. (2019).

### 2.2 Sampling and sequencing library preparation

#### 2.2.1 16S & 18S sequencing for prokarya and microbial eukarya

Vertical profiles of the ponds were sampled over a period of 13 months at intervals ranging from weekly to monthly intervals. The water was filtered through a GF/F filter (pore size 0.7 μm) and DNA extraction from the filter was conducted using a xanthogenate nucleic acid isolation (Tillett and Neilan, 2000) followed by two phenol:chloroform:isoamyl alcohol extractions, one isopropanol precipitation and one ethanol precipitation. Pellets were dissolved in pure water. The isolated DNA was stored at −20°C until further processing.

The 16S and 18S regions were PCR-amplified, resulting in 464 bp and 576 bp fragments for the 16S and 18S, respectively. The primer sequences are listed in Supplementary Table S1 (Klindworth et al., 2013; Hugerth et al., 2014) and include frameshifts by random nucleotides to enhance the sequencing performance on Illumina platforms. The PCRs were performed for 16S amplicons as follows: 1× QIAGEN Multiplex PCR Master Mix (Catalogue No. 206145) and 0.5 μM of each primer (the frameshifted primers mixed in equimolar amounts). Thermocycling conditions consisted of an initial denaturation of 95°C for 15 min; 33 cycles of 95°C for 30 s, 60°C for 30 s, 72°C for 30s; and extension of 72°C for 10 min. The PCRs were performed for 18S amplicons as follows: 1× Phusion HF buffer (Catalogue No. M0535L), 0.2 mM dNTPs (Promega Catalogue No. U151B), 0.4 μM of each primer and 0.5 U Phusion polymerase (Catalogue No. M0535L). Thermocycling conditions consisted of an initial denaturation of 98°C for 30 s; 33 cycles of 98°C for 5 s, 60°C for 30 s, 72°C for 45 s; and extension of 72°C for 5 min. PCR products were cleaned using AMPure XP beads in a ratio of 0.8× per sample. The samples were indexed using the standard Illumina Nextera XT index kit and sequenced on an Illumina MiSeq machine in a single flow cell in paired-end mode with 300 bp-read-lengths at the Genetic Diversity Center (GDC), ETH Zürich. 50,062 ± 29,087 and 3,949 ± 7,958 reads were recovered for the 16S and 18S amplicons, respectively (Supplementary Figure S1).

#### 2.2.2 eDNA sequencing targeting the COI gene for all eukarya

The eDNA samples were collected similarly and extracted with the DNeasy Blood & Tissue Kit (Qiagen, Germany) with small adaptations (Deiber et al., 2015). The amplified fragment (313 bp) targeted the COI barcode using degenerate primers that were established in previous studies (Geller et al., 2013; Leray et al., 2013). The PCR reaction consisted of SigmaFree water, 1× Buffer I (Thermo Fisher Scientific, MD, United States), BSA (0.1 mg/μL), dNTP (0.2 mM), MgCl_2_ (1 mM), forward and reverse primer (0.5 μM each), the polymerase AmpliTaq Gold (1.25 U/μL) and the DNA template as aliquot of 3 μL in a total volume of 30 μL. The PCR protocol was the following: initial denaturation at 95°C for 10 min. Thermal cycling (× 44) with denaturation of DNA at 95°C for 15 s, annealing at 62°C for 30 s, extension at 72°C for 45 s. After the last cycle, temperature remained at 72°C for 5 min, before cooling down to 10°C. The PCR products were cleaned using the Illustra GFX 96 PCR Purification Kit, indexed using the standard Illumina Nextera XT index kit and sequenced on an Illumina MiSeq machine in a single flow cell in paired-end mode with 300 bp-read-lengths at the Genetic Diversity Center (GDC), ETH Zürich. 5,886 ± 12,422 reads were recovered for the COI amplicon (Supplementary Figure S1).

### 2.3 Data analysis

#### 2.3.1 ASV/OTU calling

##### 2.3.1.1 16S & 18S

Amplicon sequence variants (ASVs) were obtained from the raw data using the nextflow (version 21.04.3) pipeline ampliseq (version 2.1.0) with default parameters, obtained from nf-core (Tommaso et al., 2017; Ewels et al., 2020; Straub et al., 2020). FastQC (version 0.11.9) was used for analyzing sequence quality (Andrews et al., 2012). Cutadapt (version 3.2) was used for trimming reads (Martin, 2011). DADA2 (version 1.20.0) was used for inferring the ASVs using Silva (138.1 prokaryotic SSU) and PR2 (Protist Reference Ribosomal Database version 4.14.0) for 16S and 18S, respectively (Guillou et al., 2013; Quast et al., 2013; Callahan et al., 2016). A mean of 50,062 and 3,949 reads were obtained for the 16S and 18S targets, respectively (Supplementary Figure S1). However, no rarefaction was done to prevent loss of data. Analyses downstream of this were done mainly in the R computing environment (version 4.1.3) using Tidyverse for data handling (Wickham et al., 2019; R Core Team, 2022).

##### 2.3.1.2 COI gene

After completion of the Illumina MiSeq run, the data quality of the demultiplexed reads was checked using FastQC (Andrews et al., 2012). Raw reads were first end-trimmed (usearch v10.0.240, R1:30nt, R2:50nt) and subsequently merged with a minimum overlap of 15 bp and a maximum overlap of 300 bp using Flash (v1.2.11). In a next step, primer sites (full-length and no mismatch) were removed from the merged reads using cutadapt (v1.12). The merged and primer trimmed reads were quality-filtered using prinseq-lite (v0.20.4) with the following parameters: size range (100–500), gc range (30–70), mean quality (Bergström et al., 2000), and low complexity filter dust (Donohue et al., 2013). The UNOISE3 (usearch v10.0.240) workflow (Edgar, 2016) with an additional clustering of 99% identity was applied to obtain error corrected and chimera filtered sequence variants (zero-radius OTUs). Sequences were checked for stop codons using invertebrate mitochondrial code. Thus, all the resulting zero-radius Operational Taxonomic Units (further referred to as zOTUs) have intact open reading frames.

#### 2.3.2 Redundancy analysis (& PERMANOVA)

Distance-based redundancy analysis (RDA) was performed on normalized amplicon counts (relative abundance) using either sampling data, treatment, and replicate or the above and oxygen, turbidity, alkalinity, conductivity, pH, total nitrogen, and total phosphorus levels as explanatory variables. The distance metric was Bray–Curtis dissimilarity. Capscale function from the R package vegan (version 2.5-7) was used (Oksanen et al., 2020).

Statistical significance of the treatment-induced differences in community composition was analyzed using permutational multivariate analysis of variance (PERMANOVA). The assumption of non-heteroscedasticity was ensured by performing a permutation test for homogeneity of multivariate dispersions; PERMANOVA was used only when dispersion was homogenous. The distance metric was Bray–Curtis distance. Adonis function from the R package vegan (version 2.5-7) was used (Oksanen et al., 2020).

#### 2.3.3 Diversity analysis

Beta diversity (Bray–Curtis and WUNIFRAC) indices were calculated using the distance function from the phyloseq package (version 1.38.0) (McMurdie and Holmes, 2013). Read counts were transformed to relative abundance prior to diversity analysis. Multiple metrics were used during the analysis to understand different aspects of the data distribution.

#### 2.3.4 Pairwise distance analysis

To study the association between environmental and community composition states in the ponds, distance was measured between every pair of samplings from the same pond, i.e., samples were compared across time only, and not across treatment or replicate. For environmental distance, pH, alkalinity, conductivity, turbidity, nitrogen, phosphorus, and oxygen levels were used as the factors defining the state of the sample’s system. For community composition distance, counts of the ASVs were used as the factors defining the state of the sample’s system. For each pair of samples taken from the same pond, the community composition variation is calculated as the Bray–Curtis distance between the samples’ percent abundance of ASVs and the environmental variation is calculated as the euclidean distance between the samples’ ecosystem properties (pH, alkalinity, conductivity, turbidity, N_2_, P, and O_2_ levels). Comparison of the two pairwise changes in the two kinds of system states allows analysis of whether the community composition changes proportionately with the environment.

#### 2.3.5 Structural equation modeling

Piecewise Structural Equation Modeling (pSEM) was used for causal hypothesis testing involving treatments, environmental variables, and community and biomass proxies (piecewiseSEM version 2.1.2) (Lefcheck, 2016). We used the Bray–Curtis distance of the community composition from the previous state as a proxy for the change in community composition at a given sampling point. Data from different sampling intervals, namely pre-disturbance, disturbance, post-disturbance, and winter were tested separately. Data from all ponds were used to test the model. Each equation within the pSEM is a linear mixed effects model with pond as a random effect and accounting for temporal auto-correlation of the dependent variable within the pond.

Our initial model consisted of experimental treatments (foundation species) affecting the community and that in turn affecting the endogenous environmental factors. The model was further tested with multiple ways of representing the interaction of the foundation species, *Myriophyllum* and *Dreissena*. After observing that the treatment effects were mainly seen post-disturbance, modeling was performed for the sampling intervals separately. Since change in the community structure from the previous state was used as a proxy for the community, each of the other biomass and ecosystem variables used were also transformed to represent change in the value from the previous state.

Missing causal links in our model, identified by tests of directed separation, were included successively and non-significant paths removed [see supporting methods in the Supplementary material for the model(s)]. When a significant path was identified that did not have any biological context to support it, the path was included as a correlation. The model with the lowest Akaike information criterion (AIC) was selected.

## 3 Results

### 3.1 Nutrient disturbance affects the trajectory of community change in prokarya but not eukarya

**Figure 2:**
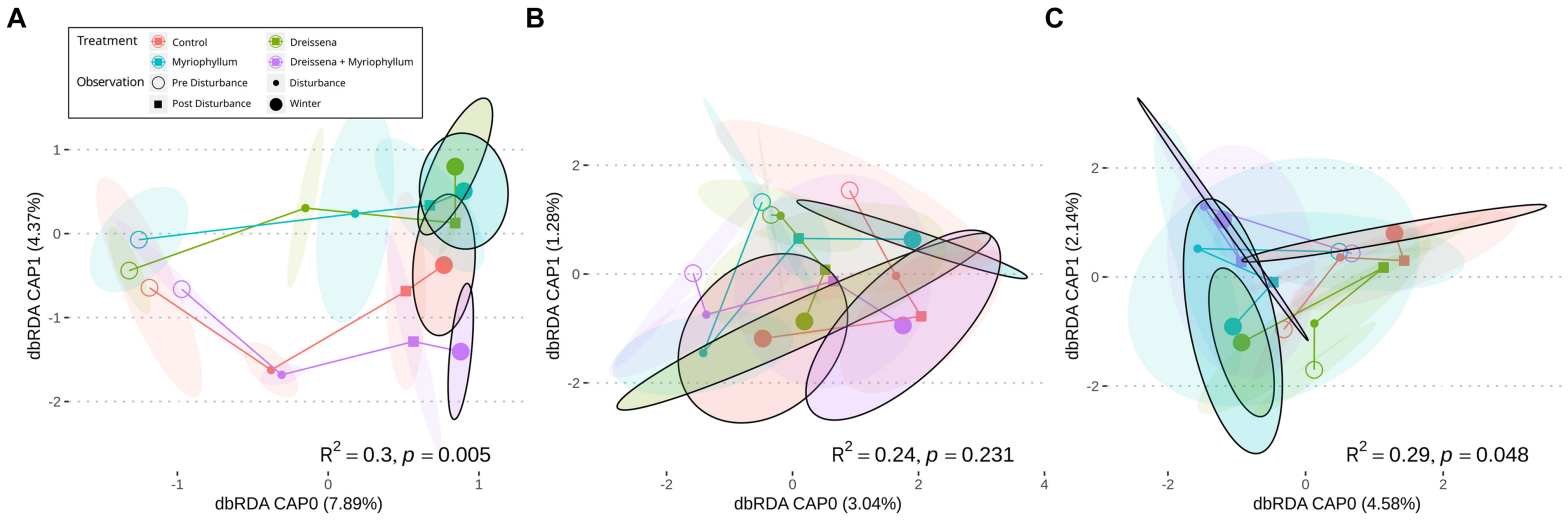
Mean community trajectory visualized after redundancy analysis (RDA) for **(A)** prokaryotes via 16S sequencing, and micro-eukaryotes via **(B)** 18S sequencing and **(C)** COI amplicon sequencing. Hollow points reflect community at start of experiment, small full point at mid-nutrient addition schedule, small full squares after nutrient addition, and big full points at the end of the sampling [8 months from the start for **(A,B)**, and 13 months for **(C)**]. The ellipses around the points represent 2 standard errors around the centroid (*n* = 4). The colors represent different treatment conditions. The *R*^2^ and *p*-values represent the separation of the differentially-treated communities at the end obtained through PERMANOVA.

**Figure 3:**
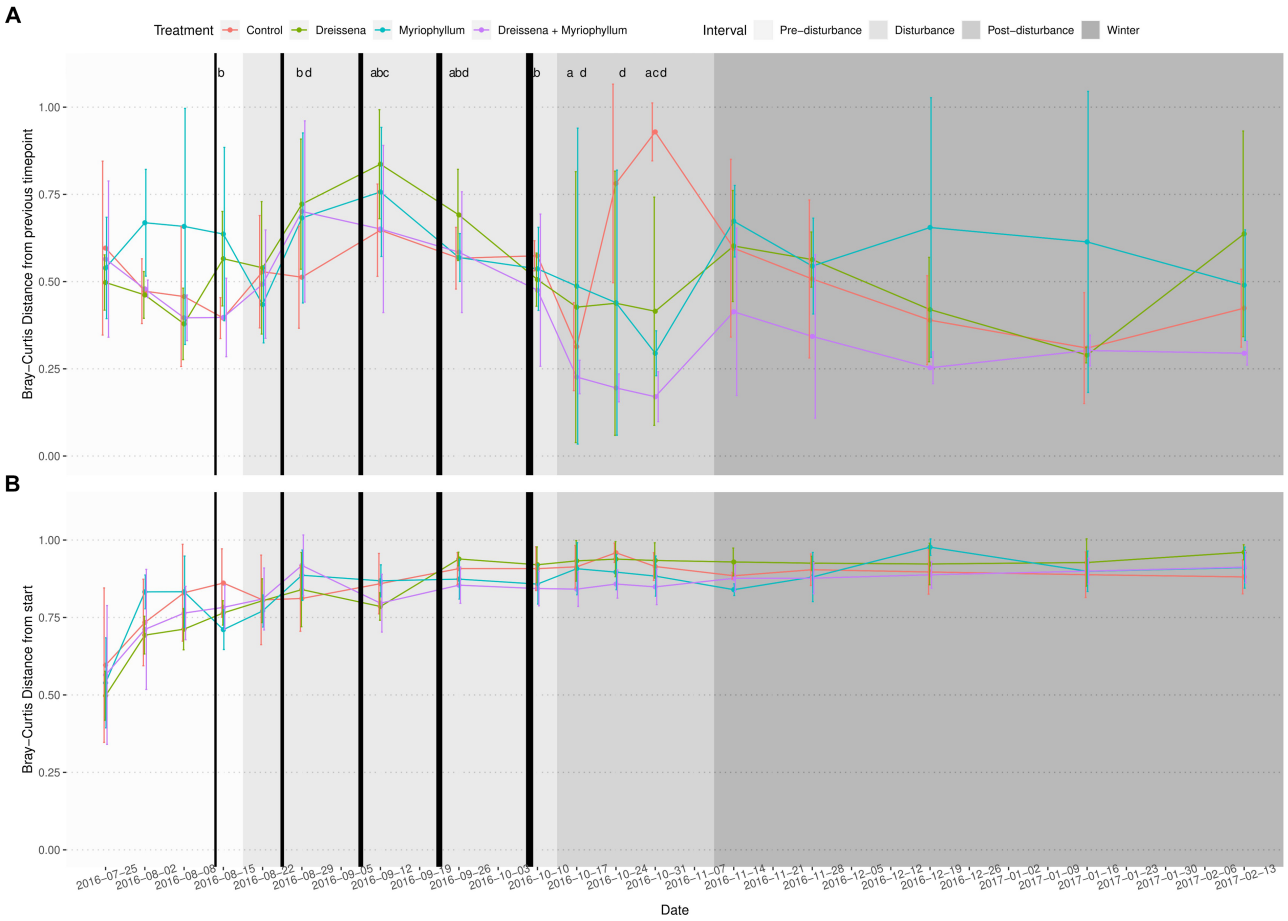
Bray–Curtis distance of the prokaryotic community in each pond from **(A)** its state at first sampling, and **(B)** its previous state (*n* = 4). The prokaryotic community displays accelerating change with increasing nutrient disturbance, which is brought to rest by the foundation species post disturbance into a new stable state. Black vertical bars represent nutrient additions, with the increasing thickness indicating increasing intensity of the additions. Letters above the lines represent significant BH adjusted *p*-values from student’s *t*-test (*p* ≤ 0.1; a = Control, b = *Dreissena*, c = *Myriophyllum*, d = *Myriophyllum* + *Dreissena*). Samples during disturbance were compared to mean pre-disturbance samples. Samples post-disturbance were compared to mid-disturbance (2016-09-12) samples.

The serial addition of nutrients in the form of nitrates and phosphates, emblematic of eutrophication, produced a strong directional shift in the prokaryotic community structure, as seen in Figure 2A, as compared to a lack of direction in the eukaryotic community structure (Figures 2B,C). The prokaryotic community moved rapidly away from its initial state at the beginning of the experiment and the change slowed down at the end of the nutrient disturbance schedule (Figure 3; Supplementary Figure S2). The rate of prokaryotic community change (measured as the dissimilarity of the current community state to the previous) increased during the nutrient disturbance compared to pre-disturbance (refer to Table 1 for the results of the *t*-tests), and then decreased post nutrient disturbance indicating a stabilizing effect of both foundation species (refer to Table 2 for the results of the *t*-tests). The community composition stability, measured as the rate of change of community composition, is disturbed again at the onset of winter, but soon re-stabilises for most treatment groups. All ponds started with abundant bacteria of the families *Comamonadaceae*, *Sphingomonadaceae*, and *Cyclobacteriaceae*. *Sphingomonodaceae* and *Spirosomaceae* grew in numbers during the nutrient disturbance, particularly in ponds with *Myriophyllum*. *Sphingomonodaceae*, *Pirellulaceae* (M and MD), and *Sporichthyaceae* (D) were abundant after the nutrient disturbance in the treatment ponds, while *Comamonadaceae* and *Sporichthyaceae* took over the non-disturbed ponds at that time (Supplementary Figure S3).

**Table 1:** Results of student’s *t*-test to check for the effect of each treatment on time points during the nutrient disturbance as compared to the mean rate of change of prokaryotic community before the nutrient disturbance, adjusted for multiple correction using the Benjamini & Hochberg method.

Date	Treatment	*t*	*p*. adj
2016-08-15	Control	−0.261	0.803
2016-08-15	*Dreissena*	3.471	**0.053**
2016-08-15	*Myriophyllum*	1.342	0.288
2016-08-15	*Dreissena* + *Myriophyllum*	0.745	0.528
2016-08-22	Control	1.500	0.260
2016-08-22	*Dreissena*	2.215	0.143
2016-08-22	*Myriophyllum*	−0.315	0.797
2016-08-22	*Dreissena* + *Myriophyllum*	1.709	0.207
2016-08-29	Control	1.435	0.268
2016-08-29	*Dreissena*	4.130	**0.030**
2016-08-29	*Myriophyllum*	1.717	0.207
2016-08-29	*Dreissena* + *Myriophyllum*	2.685	**0.095**
2016-09-12	Control	3.540	**0.049**
2016-09-12	*Dreissena*	6.563	**0.019**
2016-09-12	*Myriophyllum*	2.868	**0.076**
2016-09-12	*Dreissena* + *Myriophyllum*	2.433	0.102
2016-09-26	Control	3.402	**0.050**
2016-09-26	*Dreissena*	5.382	**0.019**
2016-09-26	*Myriophyllum*	1.880	0.187
2016-09-26	*Dreissena* + *Myriophyllum*	2.547	**0.095**
2016-10-10	Control	5.658	**0.019**
2016-10-10	*Dreissena*	4.325	**0.030**
2016-10-10	*Myriophyllum*	1.032	0.390
2016-10-10	*Dreissena* + *Myriophyllum*	1.113	0.370

Adjusted *p*-values ≤0.1 are highlighted.

**Table 2:** Results of student’s *t*-test to check for the effect of each treatment on time points after the nutrient disturbance as compared to the peak rate of change of prokaryotic community during the nutrient disturbance, adjusted for multiple correction using the Benjamini & Hochberg method.

Date	Treatment	*t*	*p*. adj
2016-10-17	Control	3.650	**0.027**
2016-10-17	*Dreissena*	1.694	0.203
2016-10-17	*Myriophyllum*	1.103	0.349
2016-10-17	*Dreissena* + *Myriophyllum*	3.472	**0.027**
2016-10-24	Control	−0.854	0.426
2016-10-24	*Dreissena*	1.686	0.203
2016-10-24	*Myriophyllum*	1.502	0.221
2016-10-24	*Dreissena* + *Myriophyllum*	3.750	**0.027**
2016-10-31	Control	−3.607	**0.027**
2016-10-31	*Dreissena*	2.030	0.168
2016-10-31	*Myriophyllum*	4.719	**0.027**
2016-10-31	*Dreissena* + *Myriophyllum*	3.845	**0.027**

Adjusted *p*-values ≤0.1 are highlighted.

The eukaryotic microbial community appeared to vary stochastically throughout the observation timeline: pre-, during, post-disturbance, and in the subsequent winter. The direction of change for the communities was arbitrary (Figure 2B) and the dissimilarity among the replicates remained high. The rate of eukaryotic community change (measured as the dissimilarity of the current community state to the previous) remained high throughout the experiment and the community did not stabilize (Supplementary Figure S4; refer to Supplementary Tables S2, S3 for the results of the *t*-tests). The eukaryotic community consisted of the families *Sessilida*, *Tetrahymenida*, and *Parameciidae* in the beginning. There were no distinctly dominant taxa among the treatments, or even among the replicates within treatments, once the nutrient disturbance began, while there were some acute irruptions of *Sessilida* in all the treatments ponds post-disturbance (Supplementary Figure S5).

The absence of signal in the eukaryotic community structure dynamics and the implied stochasticity was further confirmed by the COI amplicon data. No directionality was seen in the community composition change (Figure 2C). The COI amplicons indicated an abundance of *Brachionidae* peppered by irruptions of *Trichocercidae* and *Proalidae* throughout (Supplementary Figure S6).

### 3.2 Foundation species impose selection on prokaryotic community composition

The presence of the foundation species, *Dreissena* and *Myriophyllum*, individually and in combination, affected the trajectory of the change in the prokaryotic community composition in subtle ways as they all moved right along the CAP0 axis but differed mainly along the CAP1 axis (Figure 2A). The final compositions of prokaryotic taxa differed significantly based on the presence or absence of the foundation species [*F* = 1.53, *R*^2^ = 0.29, *p* = 0.005; permutational multivariate ANOVA (PERMANOVA)].

The trajectory of the eukaryotic community composition was not associated with treatment, as seen in the RDA path diagram (Figures 2B,C). The lack of pattern in the trajectory as well as the final composition of eukaryotic taxa is demonstrated in both the 18S and COI amplicon data (*F* = 1.29, *R*^2^ = 0.24, *p* = 0.231 and *F* = 1.10, *R*^2^ = 0.29, *p* = 0.048 respectively; PERMANOVA).

The effects of the foundation species on the communities can be observed in the relative abundance variation in the community (Supplementary Figures S3, S5, S6). The prokaryotic communities were more stable, i.e., had lower rate of change from previous compositional state, had multi-week trends, and appeared to converge to a steady state, especially in the presence of both *Dreissena* and *Myriophyllum*, while the eukaryotic communities were unstable, changed composition week-to-week, and did not appear to converge.

### 3.3 Foundation species stabilize prokaryotes’ new state post-nutrient disturbance

**Figure 4:**
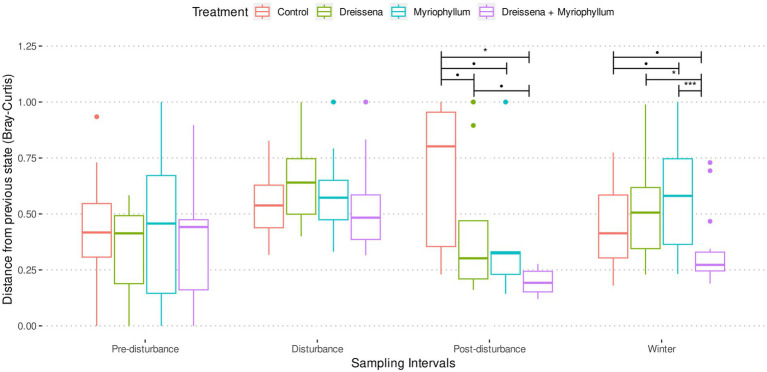
Bray–Curtis distance of the prokaryotic community in each pond from its previous state in each of the four sampling intervals. Treatment ponds cross a tipping point after nutrient additions and reach a new stable state, as inferred from slow change with respect to previous state, in contrast with control ponds. Asterisks indicate significant differences between the means as obtained from pairwise *t*-tests with Benjamini & Hochberg correction for multiple comparison.

Pre-disturbance and during disturbance, treatment had no effect on the successive change in microbial communities in the ponds (Figure 4; Supplementary Figure S7). As nutrients were added, the prokaryotic community was destabilized, changing progressively more from its previous state. At the end of the disturbance, a significant treatment effect was observed in the successive Bray–Curtis dissimilarity (*F* = 8.18, *p* = 0.0003 and *F* = 6.10, *p* = 0.0009 for post-disturbance and winter intervals resp.; ANOVA) and weighted UNIFRAC distance (*F* = 3.94, *p* = 0.012 and *F* = 5.98, *p* = 0.0017 for post-disturbance and winter intervals resp.; ANOVA).

Post-hoc analysis revealed that, post-disturbance, in ponds in which both foundation species were present (MD), the prokaryotic communities did not continue changing as quickly as the control (Figure 4; Supplementary Figure S7; pairwise *t*-tests with Benjamini & Hochberg correction for multiple comparisons). Presence of both foundation species, *Dreissena* and *Myriophyllum*, led to the greatest community composition stability, i.e., the lowest rate of change of community composition (*t* = 5.07, df = 11.65, adjusted *p* = 0.0007; *t*-test with control), while they individually stabilized the community too (*t* = 1.86, df = 21.98, adjusted *p* = 0.068 and *t* = 2.10, df = 20.97, adjusted *p* = 0.066; *t*-test with control for *Dreissena* and *Myriphyllum* present independently). This stabilizing effect of the foundation species was observed irrespective of the dissimilarity/distance metric used, whether Bray–Curtis dissimilarity (values above; Figure 4) or weighted UNIFRAC distance (*t* = 2.54, df = 11.41, adjusted *p* = 0.002, *t* = 2.73, df = 10.76, adjusted *p* = 0.002, and *t* = 3.57, df = 8.56, adjusted *p* = 0.0002; *t*-test with control for *Dreissena*, *Myriophyllum*, and both resp.; Supplementary Figure S7).

### 3.4 Presence of both foundation species led to the highest levels of turbidity and dissolved oxygen

**Figure 5:**
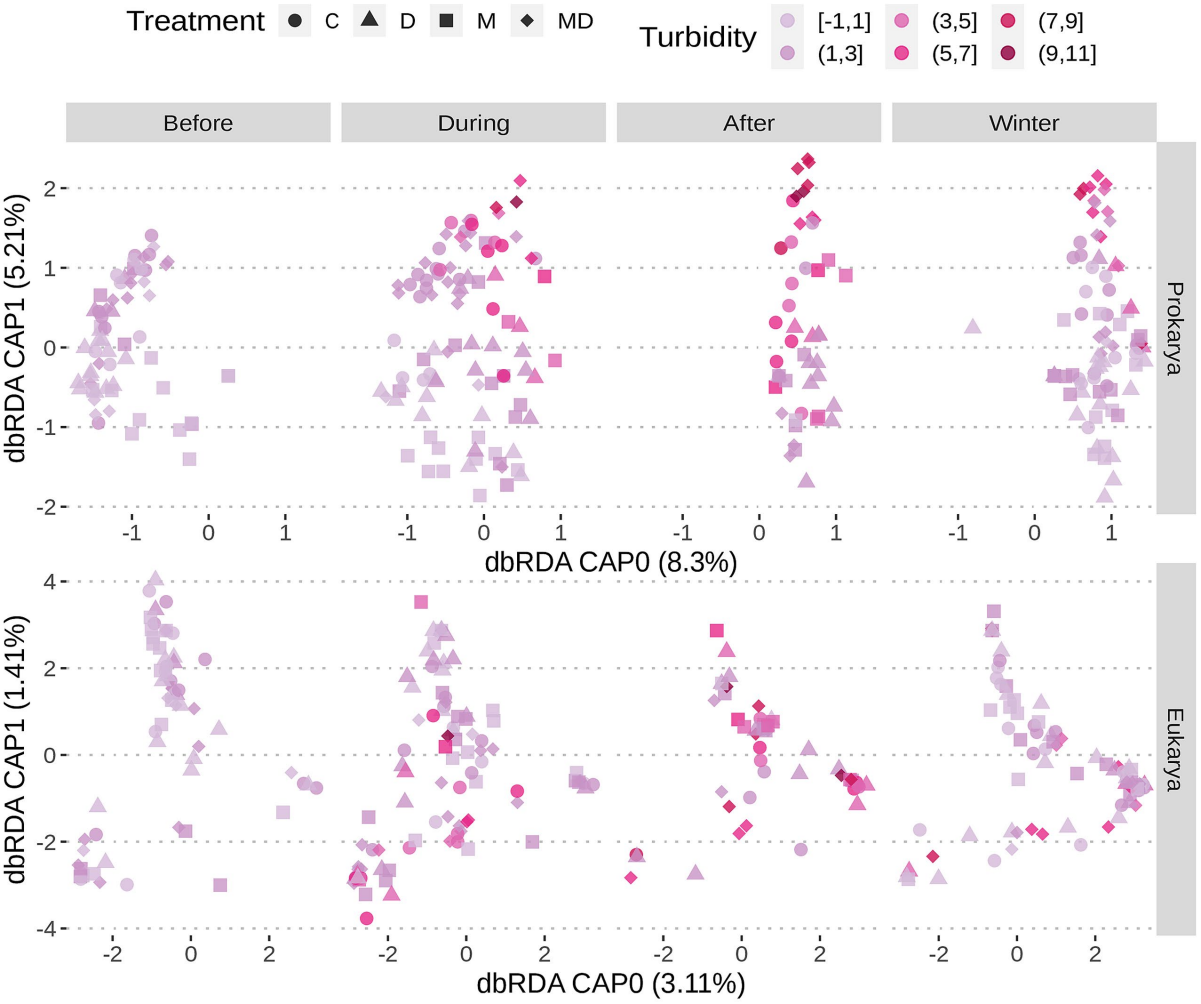
Ordination plot following redundancy analysis with samples coloured based on turbidity. Sample shapes represent treatment conditions. Samples are separated based on whether sampling was done before, during, after nutrient addition, or after onset of winter. Oxygen levels rise with nutrient additions. Prokaryotic communities in ponds with both foundation species cluster in dbRDA based on environmental variables like turbidity, while the eukaryotic communities do not.

**Figure 6:**
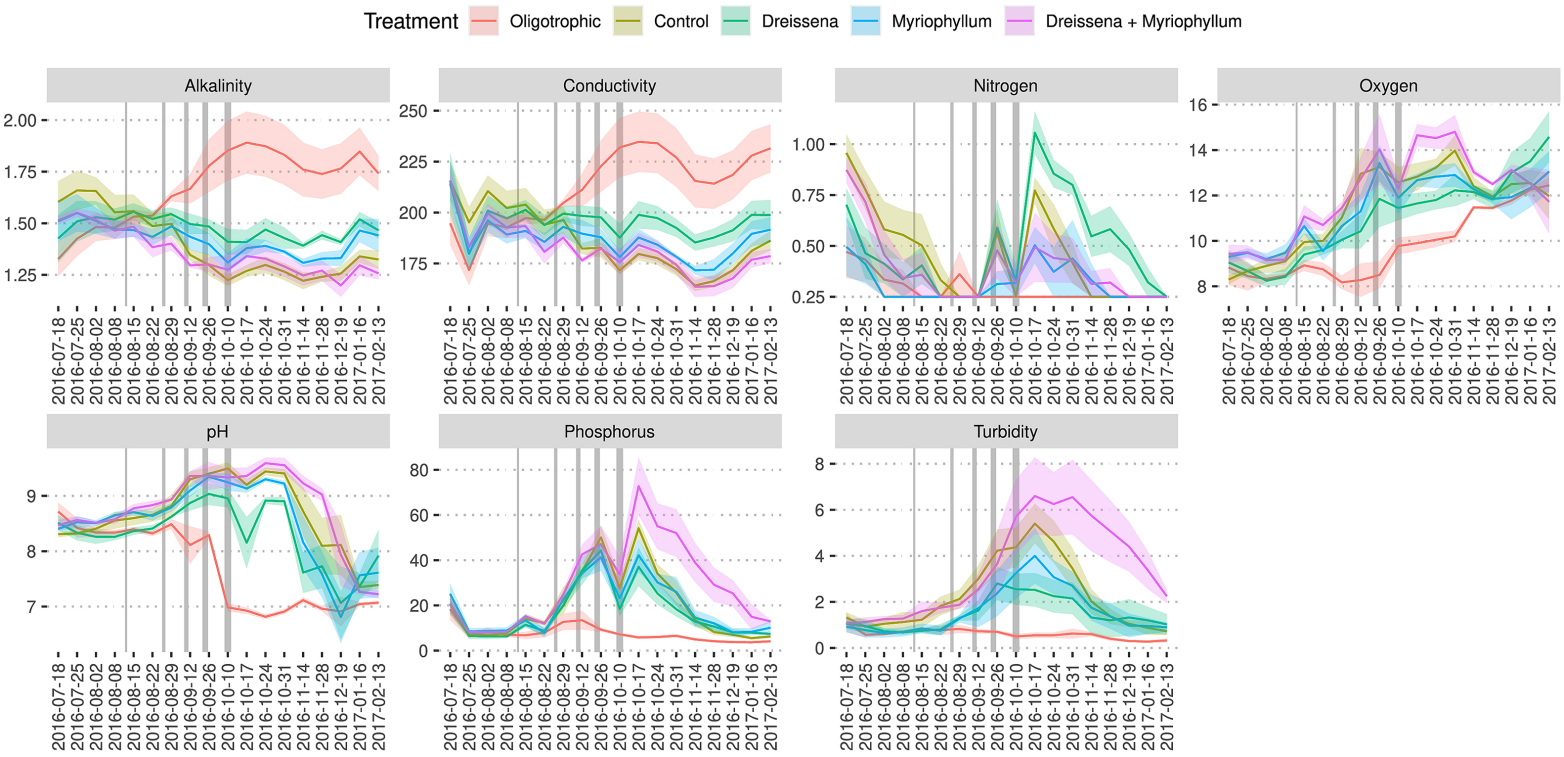
Ecosystem dynamics. Change in the treatment groups’ ponds’ alkalinity (mmol/L), conductivity (μS/cm), dissolved nitrogen level (mg/L), total oxygen level (mg/L), pH, dissolved phosphorus level (μg/cm), and turbidity (FNU). Colours indicate treatment conditions. Grey bars in the plot represent the nutrient additions. Increasing thickness of the grey bars represent the increasing nutrients in the nutrient addition schedule. Based on data from Narwani et al. (2019) and Lürig et al. (2021).

All the experimental ponds started in a clear water state with high oxygen levels (i.e., close to saturation) and progressively became turbid (Figures 5, 6; Supplementary Figure S8B) and more highly oxygenated (i.e., supersaturated) (Figure 6; Supplementary Figure S8A) throughout the nutrient addition schedule. The algal bloom, as demonstrated by the increased oxygen levels and turbidity, were observed in the ponds that received the nutrient additions, as expected, while the oligotrophic pond remained clear and maintained oxygen levels closer to that expected for the water temperature (i.e., near saturation).

Initially, the ponds were set up with low phosphorus and nitrogen levels. Over time, phosphorus levels increased steadily in the ponds that received nutrient additions, while the oligotrophic pond remained with very little phosphorus throughout the nutrient addition schedule (Figure 6). Ponds with both foundation species maintained highest levels of phosphorus, even after the nutrient additions ended. The supplemented nitrogen was absorbed by the community quickly and remained scarce throughout most of the experiment (Figure 6). The pH in the ponds started at a mildly basic condition (pH 8–9) and the ponds that received nutrients became more basic throughout the nutrient addition schedule relative to the oligotrophic ponds that decreased in pH to neutral condition (~pH 7) (Figure 6; Supplementary Figure S8C).

### 3.5 Prokaryotic community composition variation is associated with variation in ecosystem-level properties

**Figure 7:**
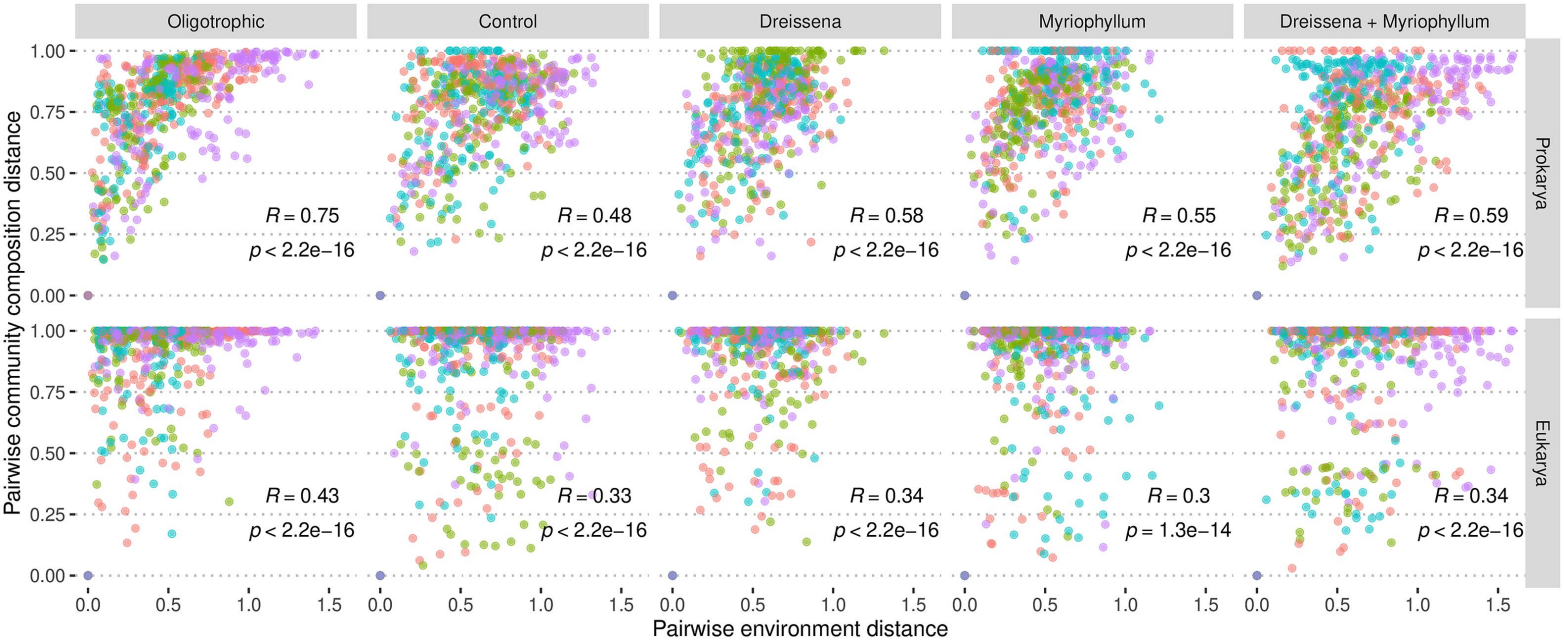
Change in community composition vs. change in environment. Change in environment is presented as the pairwise euclidean distance of the environmental covariate levels: pH, alkalinity, conductivity, turbidity, dissolved nitrogen, dissolved phosphorus, and dissolved oxygen levels. Community composition variation is presented as the pairwise Bray–Curtis distance of the percent abundance of amplicon sequence variants (ASVs). Each point represents distance between a pair of samples from the same pond, coloured by replicate number which is indicative of pond. Spearman correlation is additionally presented.

The change in community composition among the prokaryotes correlated with the change in the environmental conditions, irrespective of nutrient additions, i.e., for every pair of samples from the same pond, as the environment changed so did the corresponding prokaryotic community (Figure 7). The eukaryotic community, on the other hand, displayed a smaller correlated change.

Among the oligotrophic ponds, with neither foundation species nor nutrient disturbance, there was much higher correlation between changes in the prokaryotic communities and the environment (*R* = 0.75; Spearman correlation) as compared to changes in the eukaryotic communities and the environment (*R* = 0.43; Spearman correlation). Such a difference between the prokaryotic and eukaryotic communities’ correlation with environmental variation was also observed in ponds with either *Myriophyllum* only (*R* = 0.55 & *R* = 0.3 resp.; Spearman correlation) or both *Myriophyllum* and *Dreissena* (*R* = 0.59 & *R* = 0.34 resp.; Spearman correlation).

The environmental variation considered includes both exogenous factors that affect the community composition like nitrogen and phosphorus levels and endogenous factors that the community composition affects like oxygen levels, turbidity, pH, alkalinity, and conductivity.

### 3.6 Presence of both foundation species influences most ecosystem properties post-disturbance

Piecewise structural equation modeling (pSEM) was used to investigate the hypothesized causal relationships among the treatments, environmental variables, and community and biomass proxies. The hypothesized model was tested independently for each of the time intervals pre-disturbance, disturbance, post-disturbance, and winter; and as expected the treatment effects on the community and environment were observed only in the post-disturbance time interval. Results below elaborate on the post-disturbance model.

**Figure 8:**
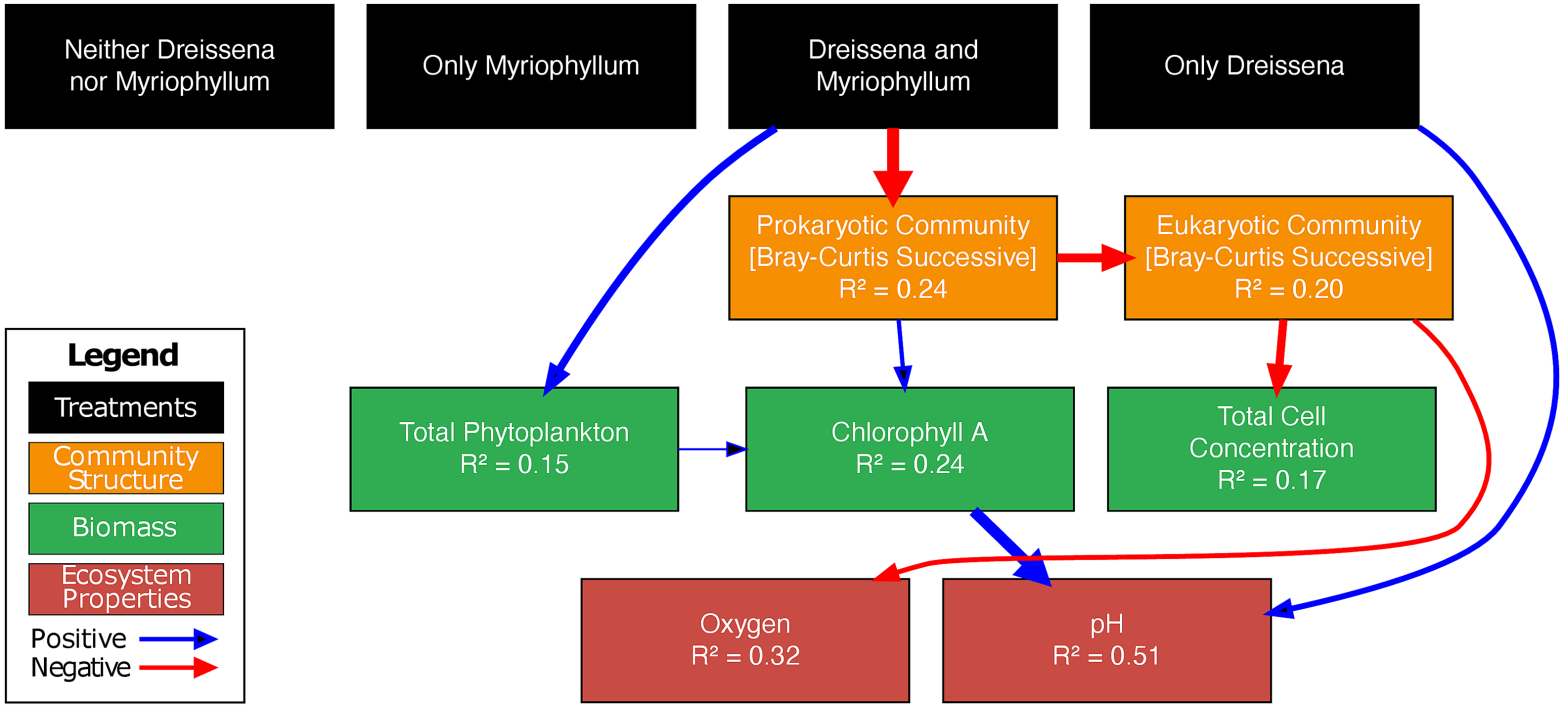
Piecewise SEM—the best fit structural equation model for the time interval post nutrient additions. Arrows represent significant causal paths, black indicating a positive relationship and red negative. Arrow thickness is proportional to its path coefficient. *R*^2^ values represent the marginal variance explained by the model. Black boxes represent experimental treatments, orange community structure, green biomass, and red endogenous ecosystem properties. For complete details of the tested model, refer to supplementary methods in the Supplementary section 1.1. The piecewise SEM model, containing pond as a random effect and including temporal autocorrelations of the 1st order (corAR1), has a Fischer’s *C* value of 72.055 with *p* = 0.476 on 72 degrees of freedom, suggesting that the model fits the data well.

The best fit model suggests that the presence of both *Dreissena* and *Myriophyllum* had a significant negative effect on the change in prokaryotic community from its previous state (measured as Bray–Curtis dissimilarity from previous state; coeff. = −0.62; Figure 8) and a significant positive effect on total phytoplankton change in the pond (measured as change in flow cytometric cell densities; coeff. = 0.39). *Dreissena* by itself affected the change in pH (coeff. = 0.30) in the pond from its previous state, while *Myriophyllum* by itself did not seem to have an effect downstream.

The prokaryotic community change directly affected the eukaryotic community change (coeff. = −0.46) and change in chlorophyll A (coeff. = 0.22). The eukaryotic community change in turn affected the change in total cell concentration (coeff. = −0.43) and oxygen levels in the pond (coeff. = −0.44). Furthermore, the total phytoplankton change in the pond had a direct positive relationship with change in chlorophyll A (coeff. = 0.47). The model most successfully explained variance in the change in pH (51%) and oxygen levels (32%).

## 4 Discussion

We investigated the independent and interactive effects of two foundation species (*Myriophyllum* and *Dreissena*) on the influence of nutrient disturbance on pro- and eukaryotic communities and the ecosystem-level functioning that they influence in artificial experimental ponds. Both foundation species and nutrient disturbances have significant effects on the trajectory and endpoints of the prokaryotic community composition, but not on the eukaryotic community composition. The change in prokaryotic community composition is associated with change in environmental variables. Post nutrient disturbance, the foundation species stabilized an alternative ecosystem state characterized by a drop in the rate of change of prokaryotic community composition and the highest amount of turbidity.

The microbial communities move away from their initial state regardless of the presence or absence of foundation species, whether it is in the form of a strong selection as in the case of prokarya or a sort of drift as in the case of eukarya (Figures 2, 3). The pressure of the monotonically increasing nutrient additions drives the ecosystems to a tipping point across which each of the treatment ponds reaches a turbid state. Following the end of the nutrient additions the ponds with single or no foundational species revert to their previous physicochemical state whereas the interactive effect of the foundation species stabilizes a new physicochemical state, characterized by a persistent turbid state (Narwani et al., 2019). We hypothesize that this stabilized community state results at least partly from the insensitivity of certain cyanobacteria to filter feeding and allelochemicals (Pimm, 1984), and is possibly mediated by positive interactions between *Dreissena* and *Myriophyllum* (Wan et al., 2022).

The foundation species used in this experiment are known to have direct and indirect effects upon the community and the environment in freshwater ecosystems (Scheffer et al., 1993; Ibelings et al., 2007; Higgins et al., 2011). Macrophytes stabilize sediments, shade phytoplankton, provide refuge for grazers, and release allelochemicals (Scheffer et al., 1993; Ibelings et al., 2007). *Dreissena* reduces suspended particulates, increases water clarity, and provides habitat structure (Ibelings et al., 2007; Higgins et al., 2011). Contrary to our expectation, both species individually stabilized the eutrophic microbial state post-nutrient additions (Figure 4), but interactively, they stabilized it even more. We hypothesize that the foundation species can interact synergistically: for instance, the presence of *Myriophyllum* could allow increased surface area for *Dreissena* to inhabit. Collaboratively, they can promote the growth of certain taxa in the community, like *Sporichythyaceae* (by *Dreissena*) and *Pirellulaceae* (by *Myriophyllum* as well as *Dreissea* and *Myriophyllum* together), while all the ponds that were nutrient disturbed were abundantly populated by *Sphingomonodaceae* and the ponds that were not by *Comamonadaceae* and *Sporichthyaceae*. Consequently, they can synergistically stabilize eutrophic community states (Pimm, 1984). However, we would also like to note that when it comes to environmental impact, it may not necessarily be the dominant constituents of the microbial community that have the greatest effect. Recent studies have shown that rare, and not abundant, microbial taxa can drive significant ecosystem effects (Chen et al., 2020; Xue et al., 2020). Consequently, it would be more appropriate to consider the complete microbial community diversity and its stability, as we have done in this study, rather than individual members of the community.

We aimed to investigate how the changes in the microbial community influenced other ecosystem functions and properties. We were interested in how changes in the prokaryotic and/or eukaryotic communities might explain changes in the total phytoplankton biomass, chlorophyll-a production, and cell concentration. In turn, because primary producers can influence both oxygen concentrations and the pH of waters, we looked at the potential for indirect impacts of the pro- and eukaryotic communities on these variables. Structural equation modeling indicated that the interactive effect of the foundation species on the changes in levels of chlorophyll-a, oxygen, and pH in the ponds was mediated by the synergistically stabilized alternative microbial community state, both prokaryotic and eukaryotic. After the nutrient additions stop, the microbial communities continue to change in the ponds with either no or only a single foundation species present, moving away from the eutrophic state with high algal concentration, characterized by lowering turbidity and phosphorus levels. Although the physicochemical state of these ponds is reverted to pre-nutrient disturbance state (Narwani et al., 2019), their bacterial and eukaryotic communities do not revert but rather continue to move away from the initial community structure. However, in the ponds with both foundational species, a turbid eutrophic state is reached post-nutrient disturbance which lasts for 3 weeks until the onset of winter. Using structural equation modeling, we find that, post-nutrient disturbance, this new stable prokaryotic community state directly stabilizes pH and indirectly stabilizes oxygen levels via the varying eukaryotic community. Our amplicon sequencing data and analysis, thus, corroborates the “critical transition or tipping point towards an alternative, turbid stable state,” proposed in Narwani et al. (2019), based on increased temporal autocorrelation in ecosystem properties in ponds with both foundation species (Dakos et al., 2019).

Strong directional pressure on the prokaryotic community in eutrophic conditions, and high variance in the eukaryotic community is consistent with other recent studies (Xue et al., 2018; Sun et al., 2020; Shabarova et al., 2021). In more extreme environments, like those with increasing salinity up to hypersaline lakes, heterotrophic bacteria and Archaea thrive while most eukaryotes die out (Elloumi et al., 2009). While it has been suggested that eukaryotic communities have low resistance but high resilience and rapid recovery via dormancy (Simon et al., 2016), it is also possible that the appearance of low resistance and the associated high variance in the community structure may be only due to lower density of eukaryotes in the local environment (Whitman et al., 1998; Caron et al., 1999; Lefranc et al., 2005; Wan et al., 2022), or because of strong top-down regulation of the small eukaryotes by cladocerans and copepods (Parslow et al., 1986; Lepère et al., 2006). Additionally, it must be considered that eukaryotic plankton communities often have a distinct separation between stable abundant sub-communities and stochastic rare sub-communities prone to experimental (sequencing noise) and biological (dormancy) artifacts (Nolte et al., 2010; Lynch and Neufeld, 2015; Xue et al., 2018).

There are certain caveats to the community analyses presented here. The apparent drift or the lack of a pattern in the eukaryotic community composition change is difficult to confirm but was aided by COI amplicon analysis corroborating the lack of pattern as seen from the 18S data. The sampling itself, although with good time resolution, was done at unequal intervals. In the winter months, change was expected to be slower and thus sampling was done less frequently, making for noisy data and gaps in the community composition state one to 2 months after nutrient additions stop. Additionally, samples were taken from multiple depths, but homogeneity across the rest of the pond is assumed. In the potential case that the abundance of eukaryotes is not homogenous and that there was a bias in their sampling, it is possible that the lack of an observed pattern in their composition change is due to lower abundance in the sampling. Finally, the amplicon sequencing only allowed high resolution up to the family taxonomic level; the genera and species of a lot of taxa were unidentifiable, making deeper analysis unfeasible.

In conclusion, we have demonstrated that the presence of foundation species, both individually and interactively, can have a significant repeatable stabilizing effect on turbid eutrophic states in aquatic ecosystems. These effects are also affected by schedule and size of nutrient additions to the system as well as climate (major effect being lower productivity in winter). Whether present as invasive species or being used in management or restoration of freshwater lakes, foundation species can have unwanted effects like stabilizing turbid eutrophic states. We recommend caution when regulating the presence of multiple foundation species, on account of possibly unanticipated interactive effects, in potentially eutrophic freshwater ecosystems to prevent prolonged algal blooms.

## Data availability statement

The datasets presented in this study can be found in online repositories. The names of the repository/repositories and accession number(s) can be found at: https://www.ncbi.nlm.nih.gov/, PRJNA1049214.

## Ethics statement

The manuscript presents research on animals that do not require ethical approval for their study.

## Author contributions

AJ: Formal analysis, Software, Visualization, Writing – original draft, Writing – review & editing. AN: Conceptualization, Data curation, Funding acquisition, Investigation, Methodology, Supervision, Writing – review & editing. BM: Conceptualization, Data curation, Investigation, Methodology, Writing – review & editing. PS: Conceptualization, Data curation, Investigation, Methodology, Writing – review & editing. JB: Data curation, Writing – review & editing. EM: Data curation, Writing – review & editing. FA: Conceptualization, Data curation, Funding acquisition, Investigation, Methodology, Writing – review & editing. MT: Formal analysis, Funding acquisition, Resources, Supervision, Writing – review & editing.
