# Supplementary material for: Foundation species stabilize an alternative eutrophic state in nutrient-disturbed ponds via selection on microbial community
Source: Front Microbiol. 2024 Apr 2;15:1310374. doi: 10.3389/fmicb.2024.1310374 (PMC11019512; doi:10.3389/fmicb.2024.1310374)
Supplement: Supplementary file 1 [file Data_Sheet_1.docx]

# Supplementary Information

## 1 Supplementary Methods

### 1.1 piecewise Structural Equation Model

**R code for the model**:

```

psem(

lme(braycurtis_bact_successive ~ M_D+nM_D+M_nD, random=~1|Pond, correlation=corAR1(form=~1|Pond), data = SEM_data, na.action = na.exclude),

lme(braycurtis_euk_successive ~ braycurtis_bact_successive, random=~1|Pond, correlation=corAR1(form=~1|Pond), data = SEM_data, na.action = na.exclude),

lme(Chl_a ~ braycurtis_bact_successive+FCMA, random=~1|Pond, correlation=corAR1(form=~1|Pond), data = SEM_data, na.action = na.exclude),

lme(TCC ~ braycurtis_euk_successive+M_D, random=~1|Pond, correlation=corAR1(form=~1|Pond), data = SEM_data, na.action = na.exclude),

lme(FCMA ~ M_D, random=~1|Pond, correlation=corAR1(form=~1|Pond), data = SEM_data, na.action = na.exclude),

lme(pH ~ Chl_a+TCC+braycurtis_bact_successive+nM_D, random=~1|Pond, correlation=corAR1(form=~1|Pond), data = SEM_data, na.action = na.exclude),

lme(Oxygen ~ braycurtis_euk_successive+FCMA, random=~1|Pond, correlation=corAR1(form=~1|Pond), data = SEM_data, na.action = na.exclude),

lme(dissolvedN ~ nM_D, random=~1|Pond, correlation=corAR1(form=~1|Pond), data = SEM_data, na.action = na.exclude),

data = SEM_data

)

```

**Summary of the model**:

```

Call:

braycurtis_bact_successive ~ M_D + nM_D + M_nD

braycurtis_euk_successive ~ braycurtis_bact_successive

Chl_a ~ braycurtis_bact_successive + FCMA

TCC ~ braycurtis_euk_successive + M_D

FCMA ~ M_D

pH ~ Chl_a + TCC + braycurtis_bact_successive + nM_D

Oxygen ~ braycurtis_euk_successive + FCMA

dissolvedN ~ nM_D

AIC BIC

168.055 238.41

---

Tests of directed separation:

Independ.Claim Test.Type DF Crit.Value P.Value

dissolvedN ~ M_D + ... coef 13 0.2784 0.7851

braycurtis_euk_successive ~ M_D + ... coef 14 0.3010 0.7678

Chl_a ~ M_D + ... coef 14 -0.7346 0.4747

pH ~ M_D + ... coef 13 1.5538 0.1442

Oxygen ~ M_D + ... coef 14 0.6575 0.5216

FCMA ~ nM_D + ... coef 13 -0.2165 0.8319

braycurtis_euk_successive ~ nM_D + ... coef 14 1.2761 0.2227

Chl_a ~ nM_D + ... coef 14 0.7650 0.4569

TCC ~ nM_D + ... coef 13 0.5852 0.5684

Oxygen ~ nM_D + ... coef 14 0.5246 0.6081

FCMA ~ M_nD + ... coef 13 0.3271 0.7488

dissolvedN ~ M_nD + ... coef 13 -0.1890 0.8530

braycurtis_euk_successive ~ M_nD + ... coef 14 -0.8410 0.4145

Chl_a ~ M_nD + ... coef 14 1.4248 0.1761

pH ~ M_nD + ... coef 13 -0.9197 0.3745

TCC ~ M_nD + ... coef 13 -0.4843 0.6362

Oxygen ~ M_nD + ... coef 14 -1.1098 0.2858

FCMA ~ braycurtis_bact_successive + ... coef 15 0.7728 0.4517

dissolvedN ~ braycurtis_bact_successive + ... coef 30 -0.5865 0.5619

TCC ~ braycurtis_bact_successive + ... coef 14 1.6140 0.1288

Oxygen ~ braycurtis_bact_successive + ... coef 13 1.2903 0.2194

dissolvedN ~ FCMA + ... coef 15 0.7114 0.4878

braycurtis_euk_successive ~ FCMA + ... coef 14 0.0154 0.9879

pH ~ FCMA + ... coef 12 -1.3531 0.2010

TCC ~ FCMA + ... coef 14 1.1120 0.2849

braycurtis_euk_successive ~ dissolvedN + ... coef 29 1.0726 0.2923

Chl_a ~ dissolvedN + ... coef 13 -2.1598 0.0500

pH ~ dissolvedN + ... coef 12 -0.8870 0.3925

TCC ~ dissolvedN + ... coef 14 -0.3448 0.7354

Oxygen ~ dissolvedN + ... coef 13 -1.3623 0.1962

Chl_a ~ braycurtis_euk_successive + ... coef 13 -1.0587 0.3090

pH ~ braycurtis_euk_successive + ... coef 12 0.2157 0.8328

TCC ~ Chl_a + ... coef 12 1.5064 0.1578

Oxygen ~ Chl_a + ... coef 12 -0.3395 0.7401

Oxygen ~ pH + ... coef 10 1.7023 0.1195

Oxygen ~ TCC + ... coef 13 1.3780 0.1915

Global goodness-of-fit:

Fisher's C = 72.055 with P-value = 0.476 and on 72 degrees of freedom

---

Coefficients:

Response Predictor Estimate Std.Error DF Crit.Value P.Value Std.Estimate

braycurtis_bact_successive M_D -0.4493 0.1685 12 -2.6656 0.0206 -0.6207 *

braycurtis_bact_successive nM_D -0.2233 0.1685 12 -1.3252 0.2098 -0.3086

braycurtis_bact_successive M_nD -0.2626 0.1703 12 -1.5419 0.1490 -0.3522

braycurtis_euk_successive braycurtis_bact_successive -0.5134 0.1460 30 -3.5161 0.0014 -0.4606 **

Chl_a braycurtis_bact_successive 10.3185 5.6290 14 1.8331 0.0881 0.2182

Chl_a FCMA 0.0000 0.0000 14 2.9869 0.0098 0.4720 **

TCC braycurtis_euk_successive -2112663.1311 882098.0030 15 -2.3950 0.0301 -0.4278 *

TCC M_D 817833.9057 723737.0990 14 1.1300 0.2775 0.2053

FCMA M_D 228625.7428 63035.7557 14 3.6269 0.0027 0.3924 **

pH Chl_a 0.0184 0.0034 13 5.3602 0.0001 0.5889 ***

pH TCC 0.0000 0.0000 13 -1.1820 0.2584 -0.1461

pH braycurtis_bact_successive 0.3310 0.1850 13 1.7892 0.0969 0.2245

pH nM_D 0.3208 0.1469 14 2.1847 0.0464 0.3007 *

Oxygen braycurtis_euk_successive -1.3724 0.3477 14 -3.9467 0.0015 -0.4369 **

Oxygen FCMA 0.0000 0.0000 14 0.4449 0.6632 0.0530

dissolvedN nM_D 52.9047 51.6865 14 1.0236 0.3234 0.1057

Signif. codes: 0 '***' 0.001 '**' 0.01 '*' 0.05

---

Individual R-squared:

Response method Marginal Conditional

braycurtis_bact_successive none 0.24 0.24

braycurtis_euk_successive none 0.20 0.34

Chl_a none 0.24 0.40

TCC none 0.17 0.17

FCMA none 0.15 0.29

pH none 0.51 0.51

Oxygen none 0.32 0.56

dissolvedN none 0.01 0.01

```

## 2 Supplementary Tables

**Supplementary Table 1**: Primer sequences

| 16S-F_nex0 | TCGTCGGCAGCGTCAGATGTGTATAAGAGACAGGACCTACGGGNGGCWGCAG |
| --- | --- |
| 16S-F_nex1 | TCGTCGGCAGCGTCAGATGTGTATAAGAGACAGNGACCTACGGGNGGCWGCAG |
| 16S-F_nex2 | TCGTCGGCAGCGTCAGATGTGTATAAGAGACAGNNGACCTACGGGNGGCWGCAG |
| 16S-F_nex3 | TCGTCGGCAGCGTCAGATGTGTATAAGAGACAGNNNGACCTACGGGNGGCWGCAG |
| 16S-R_nex0 | GTCTCGTGGGCTCGGAGATGTGTATAAGAGACAGCAGACTACHVGGGTATCTAATCC |
| 16S-R_nex1 | GTCTCGTGGGCTCGGAGATGTGTATAAGAGACAGNCAGACTACHVGGGTATCTAATCC |
| 16S-R_nex2 | GTCTCGTGGGCTCGGAGATGTGTATAAGAGACAGNNCAGACTACHVGGGTATCTAATCC |
| 16S-R_nex3 | GTCTCGTGGGCTCGGAGATGTGTATAAGAGACAGNNNCAGACTACHVGGGTATCTAATCC |
| mICOIintF | TGGWACWGGWTGAACWGTWTAYCCYCC |
| 18S-F_nex0 | TCGTCGGCAGCGTCAGATGTGTATAAGAGACAGGACGGTAATTCCAGCTCYV |
| 18S-F_nex1 | TCGTCGGCAGCGTCAGATGTGTATAAGAGACAGNGACGGTAATTCCAGCTCYV |
| 18S-F_nex2 | TCGTCGGCAGCGTCAGATGTGTATAAGAGACAGNNGACGGTAATTCCAGCTCYV |
| 18S-F_nex3 | TCGTCGGCAGCGTCAGATGTGTATAAGAGACAGNNNGACGGTAATTCCAGCTCYV |
| 18S-R_nex0 | GTCTCGTGGGCTCGGAGATGTGTATAAGAGACAGCACCGTCAATTHCTTYAART |
| 18S-R_nex1 | GTCTCGTGGGCTCGGAGATGTGTATAAGAGACAGNCACCGTCAATTHCTTYAART |
| 18S-R_nex2 | GTCTCGTGGGCTCGGAGATGTGTATAAGAGACAGNNCACCGTCAATTHCTTYAART |
| 18S-R_nex3 | GTCTCGTGGGCTCGGAGATGTGTATAAGAGACAGNNNCACCGTCAATTHCTTYAART |
| jgHCO2198 | TAIACYTCIGGRTGICCRAARAAYCA |

**Supplementary Table 2**: Results of Student’s t-test to check for the effect of each treatment on time points during the nutrient disturbance as compared to the mean rate of change of microbial eukaryotic community before the nutrient disturbance, adjusted for multiple correction using the Benjamini & Hochberg method. Adjusted p values <= 0.1 are highlighted.

Date Treatment t df p p.adj

1 2016-08-15 Control 9.7072254 6 6.859718e-05 **0.0007394082**

2 2016-08-15 Dreissena 0.9529347 5 3.843851e-01 0.4855390258

3 2016-08-15 Myriophyllum 1.1087086 6 3.100079e-01 0.4571442614

4 2016-08-15 Dreissena + Myriophyllum 1.0747120 6 3.238105e-01 0.4571442614

5 2016-08-22 Control 4.2769169 6 5.223252e-03 **0.0156697567**

6 2016-08-22 Dreissena 11.3611912 5 9.242603e-05 **0.0007394082**

7 2016-08-22 Myriophyllum 5.6169835 6 1.359477e-03 **0.0054379095**

8 2016-08-22 Dreissena + Myriophyllum 3.5029487 6 1.278044e-02 **0.0306730536**

9 2016-08-29 Control 0.5126403 6 6.265223e-01 0.7160255127

10 2016-08-29 Dreissena 4.4899642 6 4.148057e-03 **0.0142219110**

11 2016-08-29 Myriophyllum 6.4950846 6 6.337824e-04 **0.0038026946**

12 2016-08-29 Dreissena + Myriophyllum 3.1289357 5 2.598863e-02 **0.0567024731**

13 2016-09-12 Control 9.5462688 6 7.543154e-05 **0.0007394082**

14 2016-09-12 Dreissena 2.5973196 5 4.840614e-02 **0.0893651800**

15 2016-09-12 Myriophyllum 0.3465877 6 7.407326e-01 0.7663665946

16 2016-09-12 Dreissena + Myriophyllum 2.7998464 6 3.117026e-02 **0.0623405147**

17 2016-09-26 Control 3.9135610 6 7.860072e-03 **0.0209601932**

18 2016-09-26 Dreissena 0.9720125 6 3.685833e-01 0.4855390258

19 2016-09-26 Myriophyllum 1.7139729 6 1.373634e-01 0.2354801258

20 2016-09-26 Dreissena + Myriophyllum 1.2814431 6 2.473305e-01 0.3957288356

21 2016-10-10 Control 0.4551321 5 6.680949e-01 0.7288308210

22 2016-10-10 Dreissena 0.6284727 6 5.528507e-01 0.6634207949

23 2016-10-10 Myriophyllum 5.7916661 6 1.160088e-03 **0.0054379095**

24 2016-10-10 Dreissena + Myriophyllum 0.3109383 6 7.663666e-01 0.7663665946

**Supplementary Table 3:** Results of Student’s t-test to check for the effect of each treatment on time points after the nutrient disturbance as compared to the peak rate of change of microbial eukaryotic community during the nutrient disturbance, adjusted for multiple correction using the Benjamini & Hochberg method. Adjusted p values <= 0.1 are highlighted.

Date Treatment t df p p.adj

1 2016-10-17 Control 1.7360361 6 0.13323857 0.5019365

2 2016-10-17 Dreissena -1.6146275 5 0.16731215 0.5019365

3 2016-10-17 Myriophyllum 0.7227758 5 0.50220047 0.7533007

4 2016-10-17 Dreissena + Myriophyllum 1.1856374 6 0.28058752 0.5611750

5 2016-10-24 Control 1.6062306 6 0.15934652 0.5019365

6 2016-10-24 Dreissena 0.4255219 5 0.68815001 0.8228518

7 2016-10-24 Myriophyllum -0.2338829 6 0.82285177 0.8228518

8 2016-10-24 Dreissena + Myriophyllum -0.8070247 6 0.45045021 0.7533007

9 2016-10-31 Control 3.3289592 6 0.01582715 0.1899258

10 2016-10-31 Dreissena 0.5645830 5 0.59674431 0.7956591

11 2016-10-31 Myriophyllum -0.2915187 6 0.78047063 0.8228518

12 2016-10-31 Dreissena + Myriophyllum -1.3350176 6 0.23029008 0.5526962

## 3 Supplementary Figures


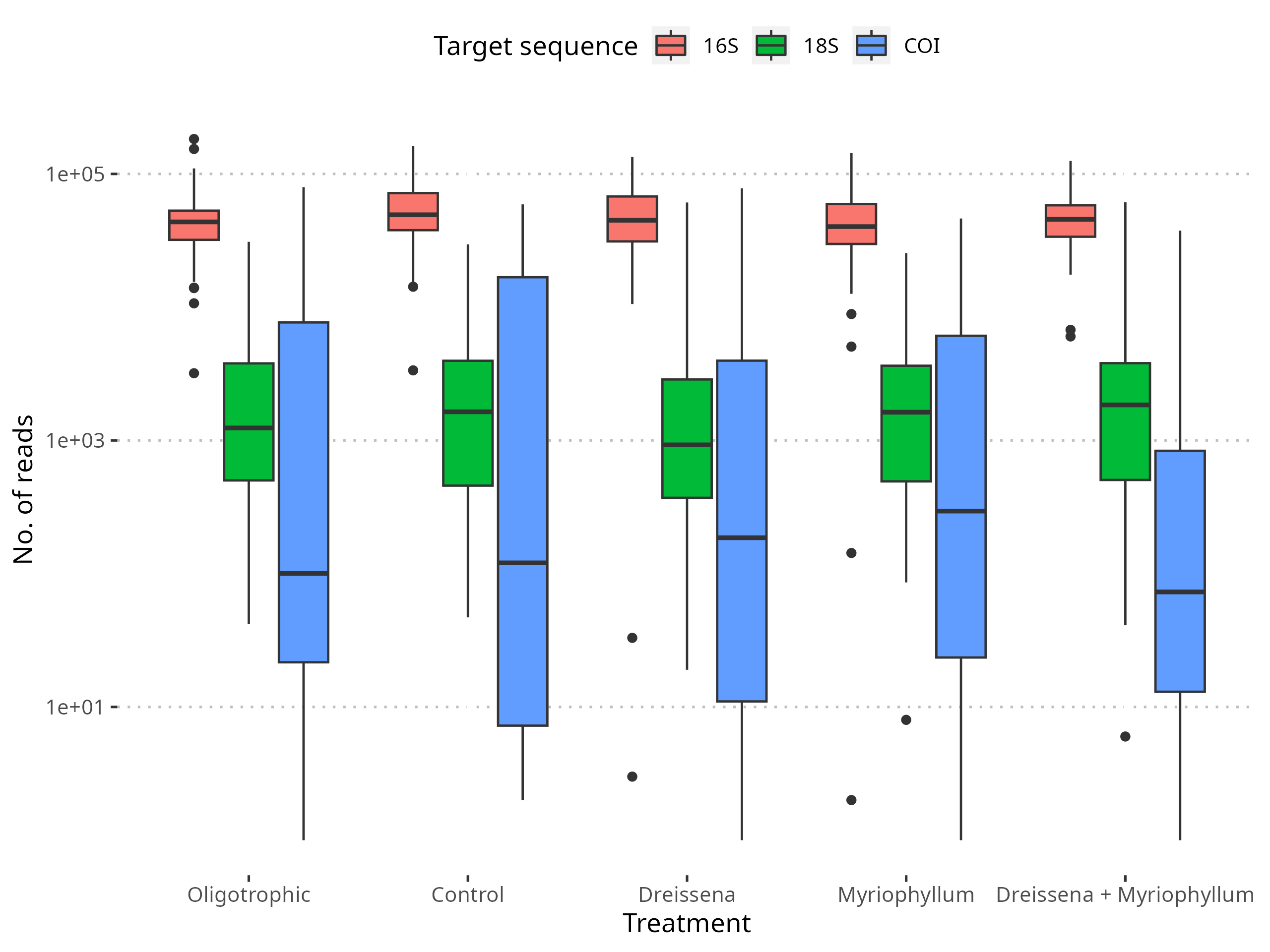
**Suppl. Fig. 1:** Read counts for the different target sequences; 16S for prokarya, and 18S and COI for eukarya. Reads from the COI sequencing were filtered to remove macro-eukaryotes before generating this plot and all downstream analysis.


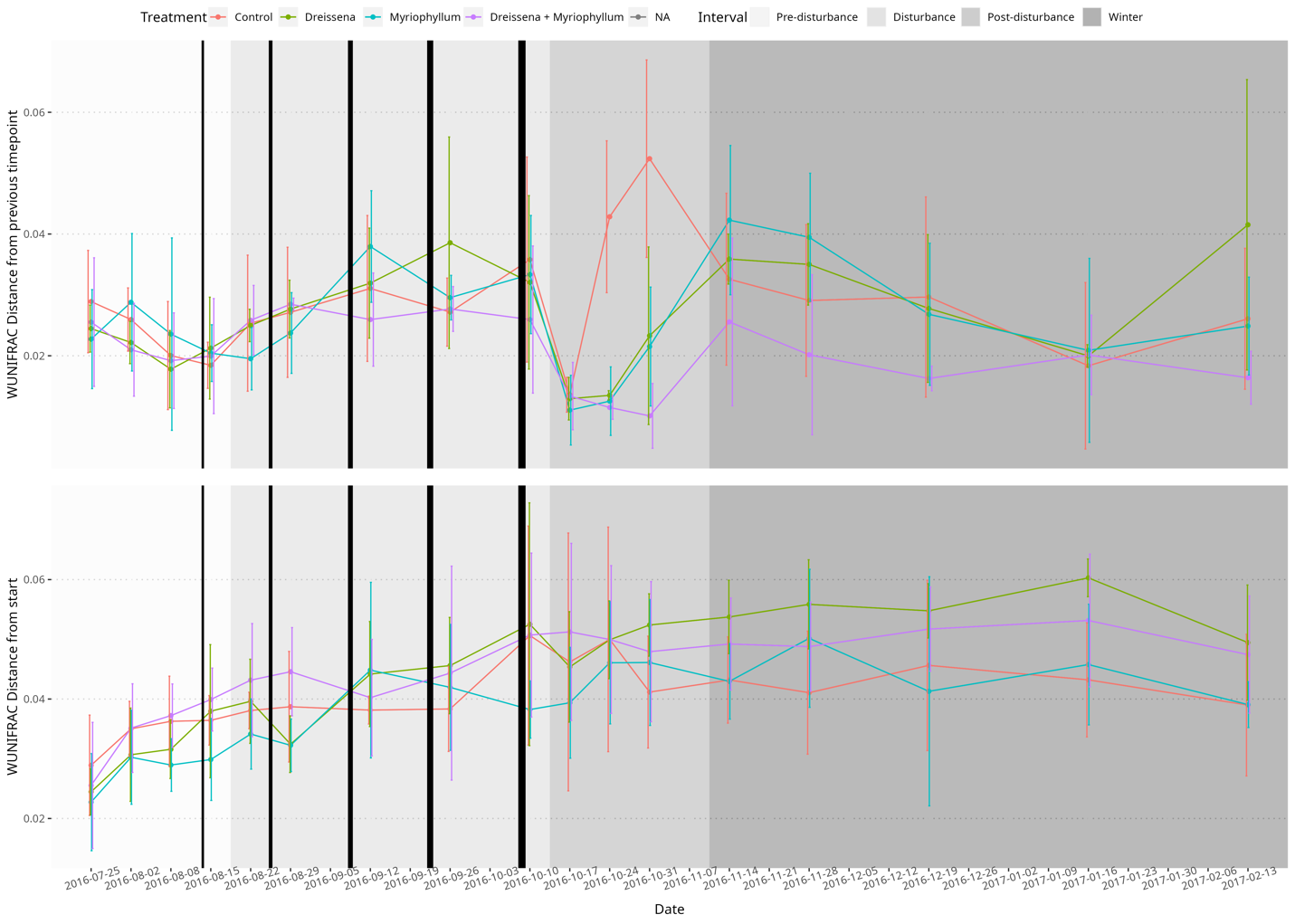


**Suppl. Fig. 2:** Weighted UNIFRAC distance of the prokaryotic community in each pond from (a) its initial state, and (b) its previous state (n=4). The prokaryotic community displays accelerating change with increasing nutrient disturbance, which is brought to rest by the foundation species post disturbance into a new stable state. Black vertical bars represent nutrient additions, with the increasing thickness indicating increasing intensity of the additions.


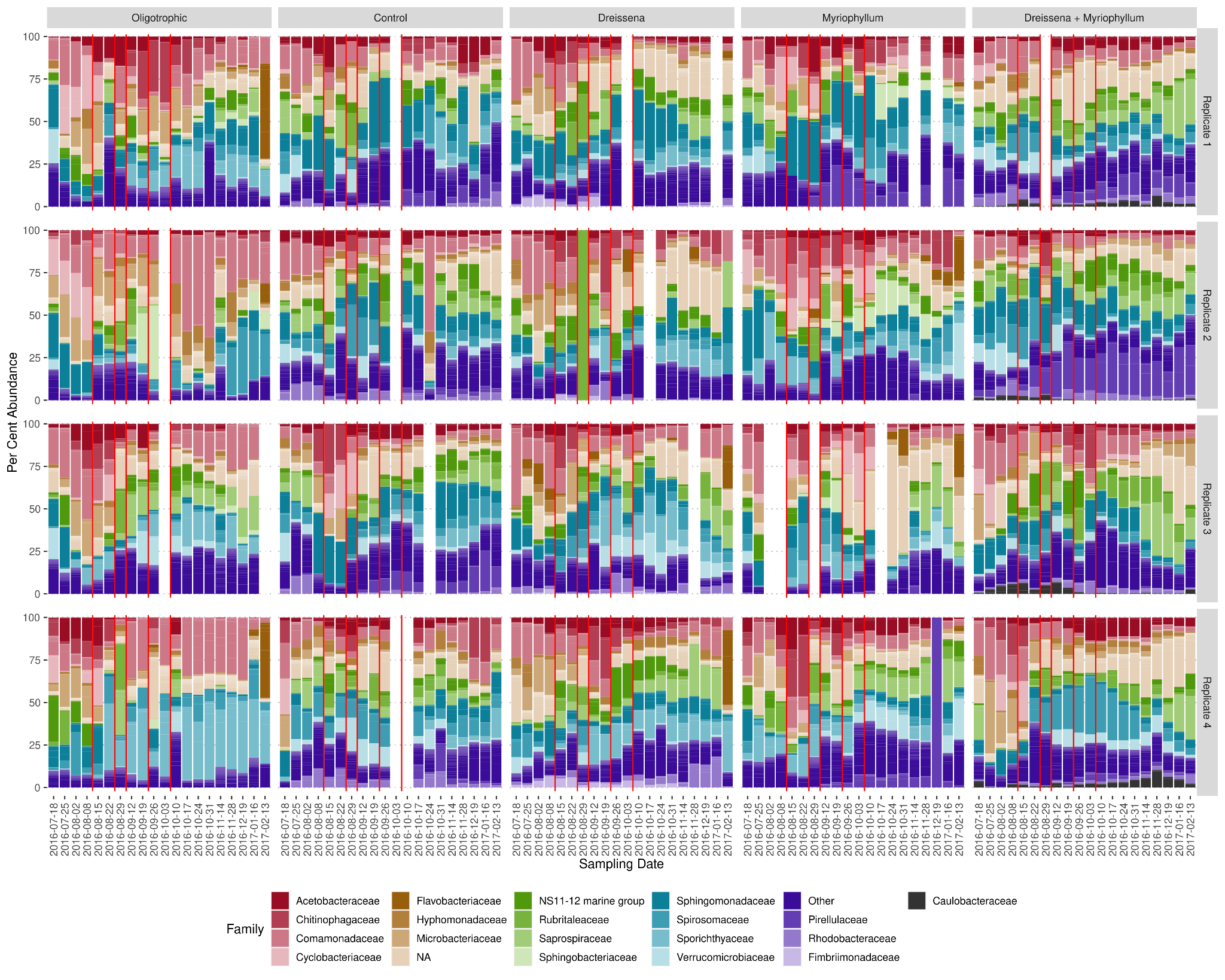


**Suppl. Fig. 3:** Relative abundance of taxonomic families in the prokaryotic community for each replicate on every sampling date, separated by treatment condition. This data was obtained from 16S ASV inference. Red lines in the chart reflect the nutrient addition schedule.


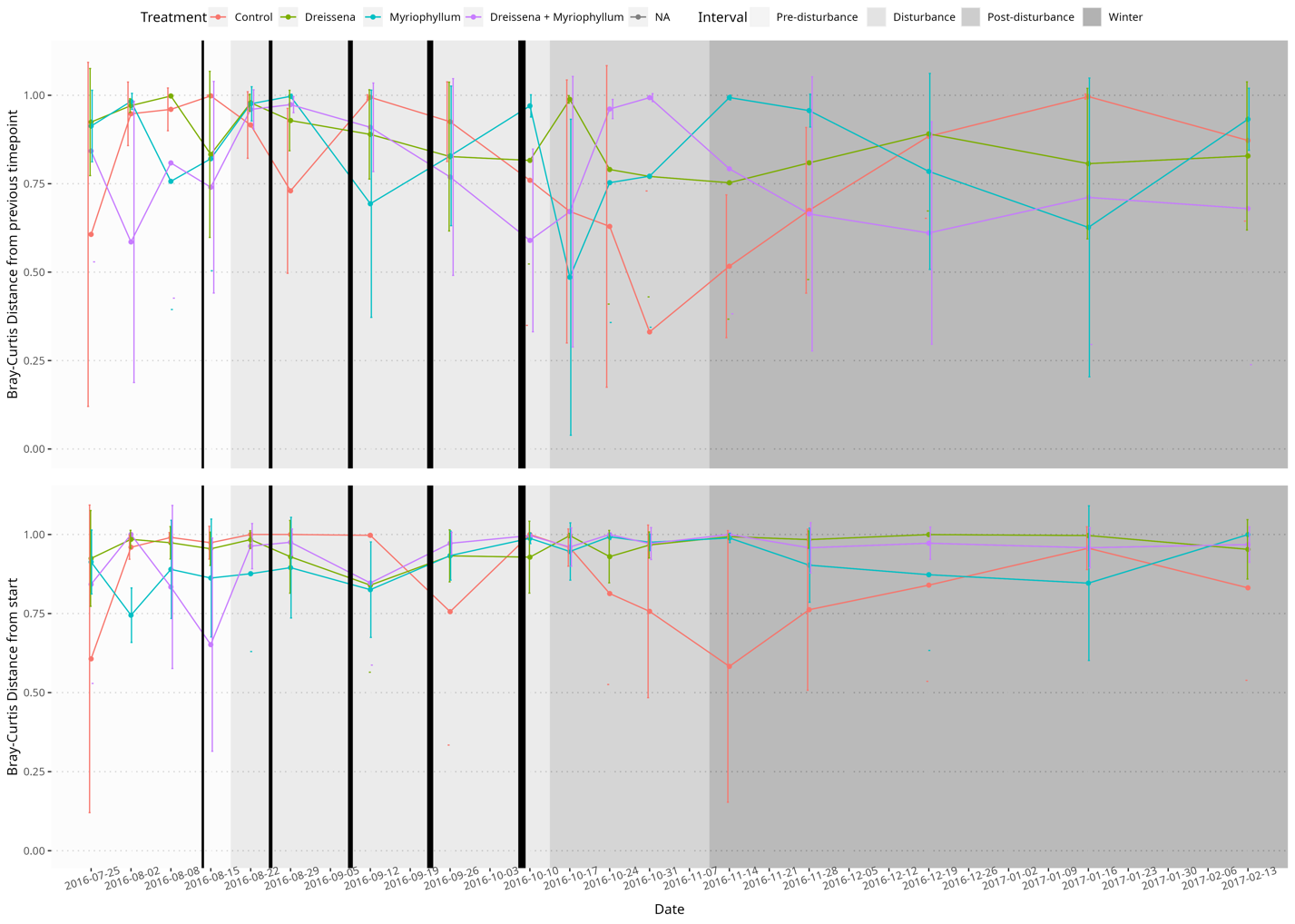


**Suppl. Fig. 4:** Bray-Curtis distance of the eukaryotic community in each pond from (a) its initial state, and (b) its previous state (n=4). The eukaryotic community continues to change week after week irrespective of treatment groups or pre-, during, post-nutrient additions, or after onset of winter. Black vertical bars represent nutrient additions, with the increasing thickness indicating increasing intensity of the additions.


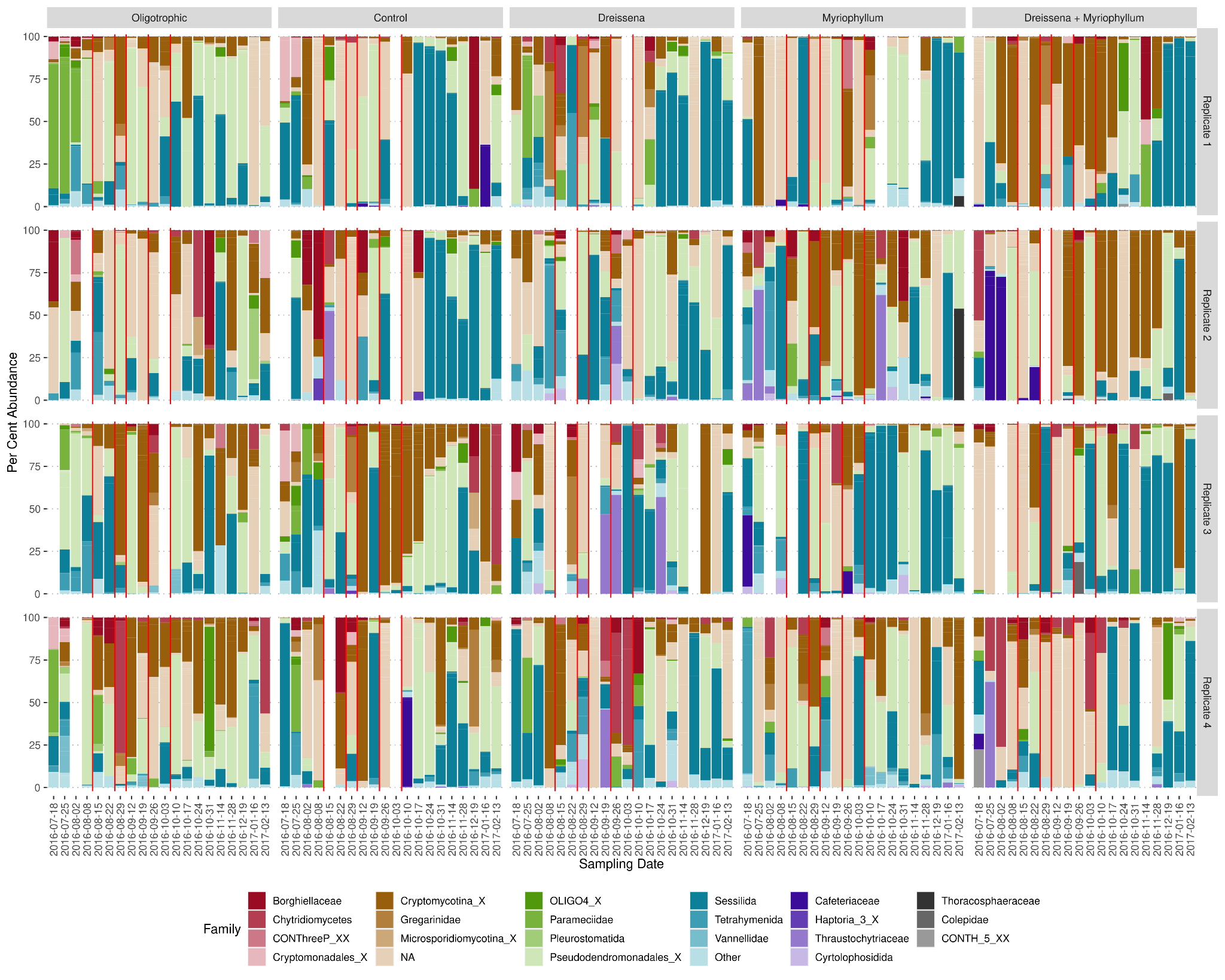


**Suppl. Fig 5:** Relative abundance of taxonomic families in the eukaryotic community for each replicate on every sampling date, separated by treatment condition. This data was obtained from 18S ASV inference. Red lines in the chart reflect the nutrient addition schedule.


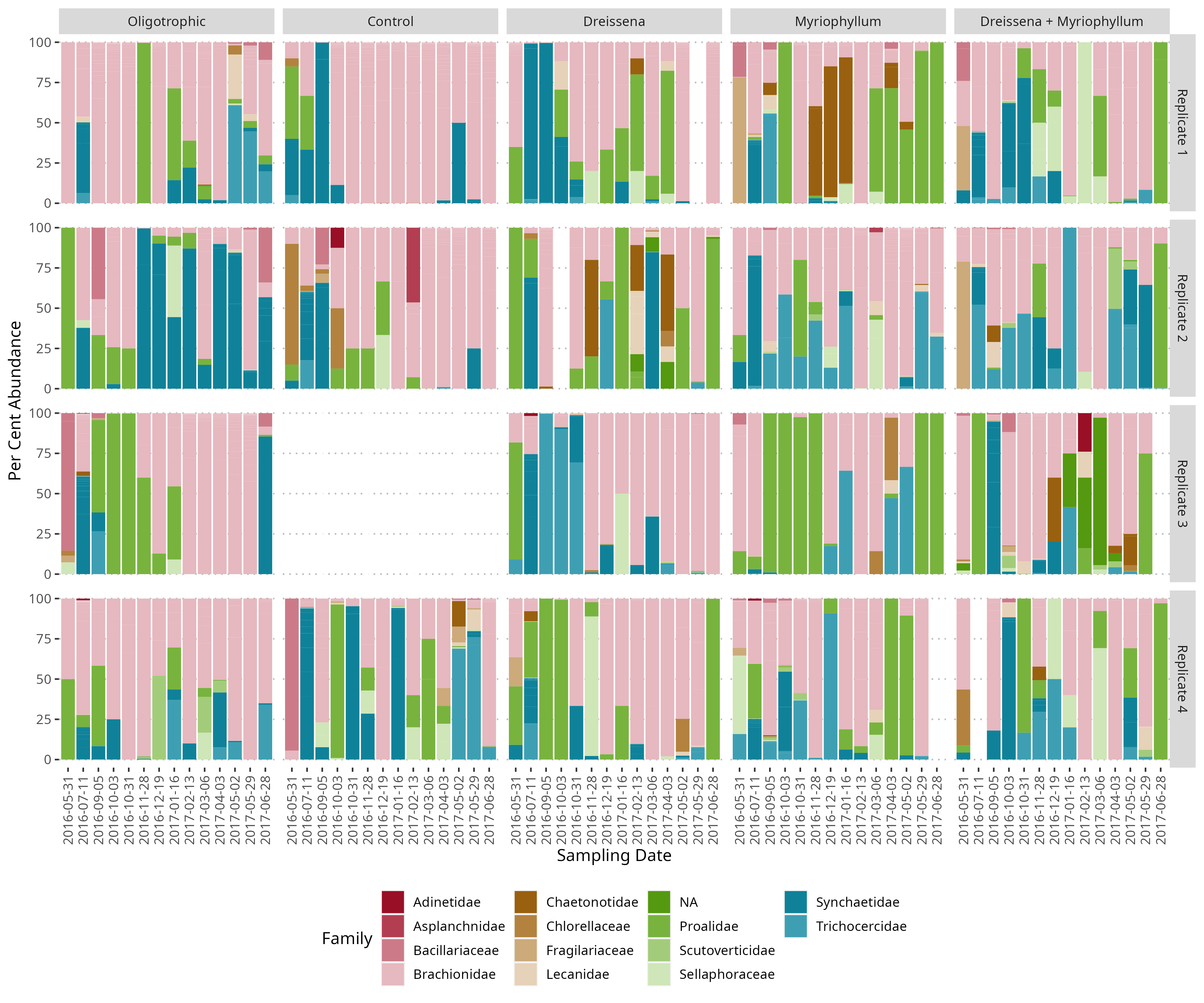


**Suppl. Fig. 6:** Relative abundance of taxonomic families in the micro-eukaryotic community for each replicate on every sampling date, separated by treatment condition. This data was obtained from OTU inference based on COI gene amplification and sequencing.


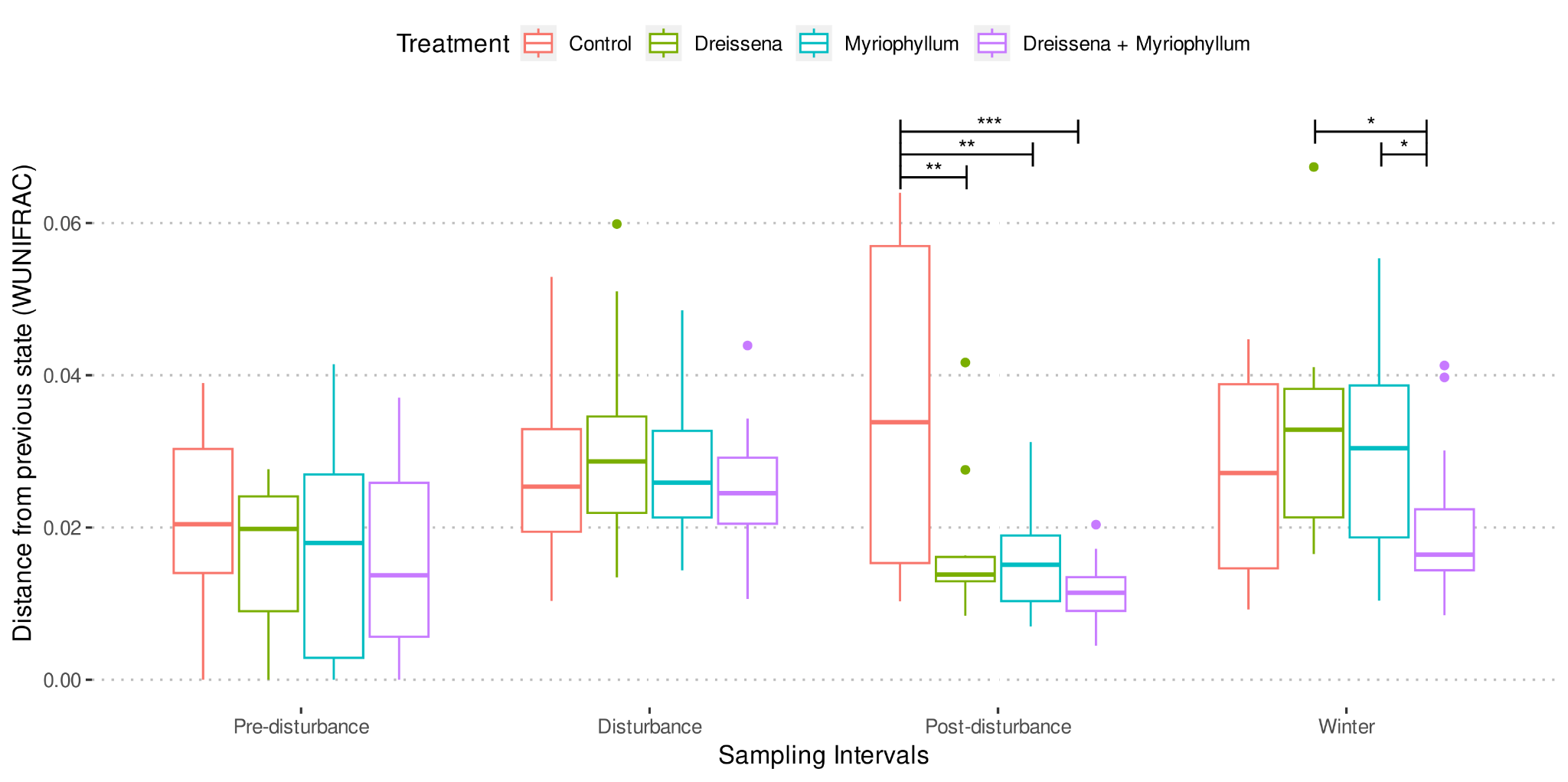


**Suppl. Fig. 7:** Weighted UNIFRAC distance of the prokaryotic community in each pond from its previous state in each of the four sampling intervals. Treatment ponds cross a tipping point after nutrient additions and reach a new stable state in contrast with control ponds. Asterisks indicate significant differences between the means as obtained from pairwise t-tests with Benjamini & Hochberg correction for multiple comparisons.


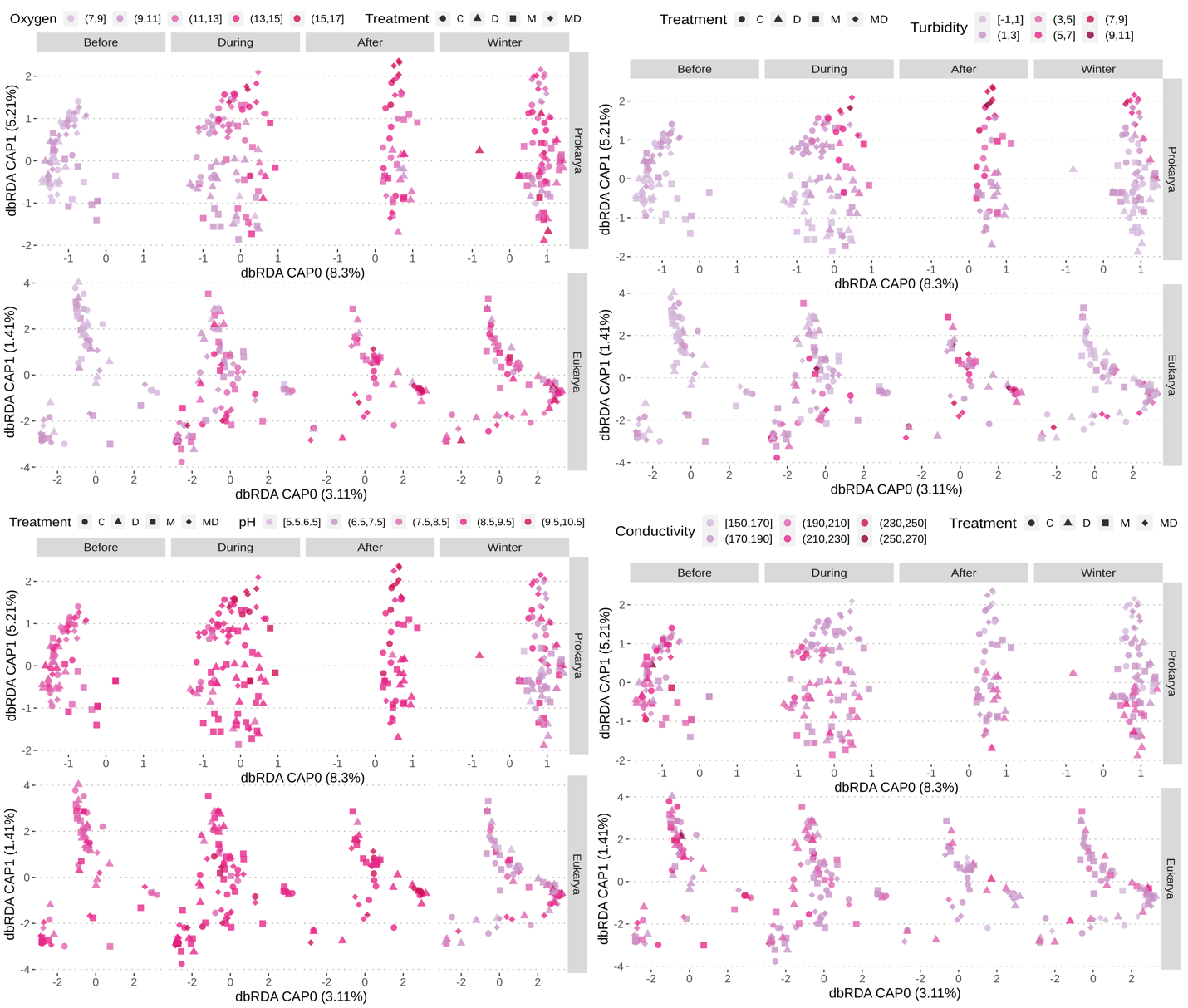
**Suppl. Fig. 8:** Ordination plot following redundancy analysis with samples coloured based on (a) oxygen (*mg/L*), (b) turbidity (*FNU*), (c) pH levels, and (d) Conductivity (*μS/cm*). Sample shapes represent treatment conditions. Samples are separated based on whether sampling was done before, during, after nutrient addition, or after onset of winter
